# Missing Excitons: How Energy Transfer Competes with Free Charge Generation in Dilute-Donor/Acceptor Systems

**DOI:** 10.1021/acsenergylett.3c01969

**Published:** 2024-02-08

**Authors:** Joshua M. Carr, Melissa K. Gish, Obadiah G. Reid, Garry Rumbles

**Affiliations:** † Materials Science and Engineering Program, University of Colorado Boulder, Boulder, Colorado 80303, United States; ‡ Chemistry and Nanoscience Center, National Renewable Energy Laboratory, Golden, Colorado 80401, United States; § Renewable and Sustainable Energy Institute, University of Colorado Boulder, Boulder, Colorado 80303, United States; ∥ Department of Chemistry, University of Colorado Boulder, Boulder, Colorado 80303, United States

## Abstract

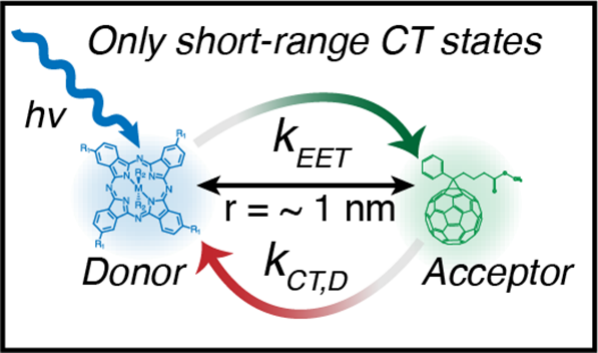

Energy transfer across the donor–acceptor interface in organic photovoltaics is usually beneficial to device performance, as it assists energy transport to the site of free charge generation. Here, we present a case where the opposite is true: dilute donor molecules in an acceptor host matrix exhibit ultrafast excitation energy transfer (EET) to the host, which suppresses the free charge yield. We observe an optimal photochemical driving force for free charge generation, as detected via time-resolved microwave conductivity (TRMC), but with a low yield when the sensitizer is excited. Meanwhile, transient absorption shows that transferred excitons efficiently produce charge-transfer states. This behavior is well described by a competition for the excited state between long-range electron transfer that produces free charge and EET that ultimately produces only localized charge-transfer states. It cannot be explained if the most localized CT states are the intermediate between excitons and the free charge in this system.

Recent advances in organic photovoltaics (OPVs) due to nonfullerene acceptor molecules has generated a resurgence in the field, with bulk heterojunction (BHJ) OPVs reaching 19% power conversion efficiencies.^1^ However, the underlying photophysics of how organic semiconductors separate excitons into free charges is still debated, with many models in the literature suggesting a variety of possible explanations.^2−11^ We recently introduced a new model to explain free charge generation in organic photovoltaics that we call distributed-range electron transfer (DRET), because it invokes a distribution of charge-transfer distances. Charge separation is described as a competition between short-range, localized charge-transfer (CT) states (*<*ca. 3 nm) with large reorganization energy and long-range, delocalized free-charge (FC) states (*>*ca. 3 nm) with small reorganization energy. In each case we use a Marcus rate expression and an integration over the available density of states to calculate the overall rate constants.^12^ Crucially, the CT states are posited to be too tightly bound to further dissociate and are not an intermediate to the FC states. This DRET model quantitatively describes the normal, optimal, and inverted regimes we observe in dilute donor–acceptor systems and explains why the inverted regime is rarely observed in qualitatively similar photocurrent measurements on OPV devices.^13^

Here, we investigate the role that energy transfer plays as a competing pathway in dilute donor–acceptor systems, both as a test of the DRET model and as a way of deepening our understanding of energy-transfer processes in organic photovoltaics. A crucial feature of the molecular system in much of our prior work was the ability to selectively excite the donor species at excitation energies lower than the acceptor (nominally called the “red sensitizers”), which simplifies the kinetics of the system, not allowing for any competing pathways, such as energy transfer.^12,14,15^ In the present work, we instead examine a series of molecular donors sensitizing a 6,6-phenyl C_61_-butyric acid methyl ester (PCBM) host, but at excitation energies slightly higher than the PCBM host (nominally called the “blue sensitizers”). This change in excitation energy opens an energy-transfer pathway that competes with charge transfer. Surprisingly, the slight shift in energetics has a profound influence on the outcome. In what follows we show that selective excitation of these “blue sensitizers” results in little if any free charge but efficiently produces localized charge-transfer states. While these findings may seem contrary to much prior work showing that energy transfer benefits OPV device performance,^16−18^ the difference in charge-transfer entropy in bulk hetrojunction blends relative to our dilute sensitized systems^5^ suggests phenomena like that reported here will only dominate in too finely intermixed regions of such a device and that energy-transfer processes will *usually* be beneficial.

We report free charge yield as a function of Gibbs energy for photoinduced electron transfer,^19^ Δ*G*_*CT*_, (eq 1) in a series of “blue donors” sensitized into a PCBM accepting host matrix. In contrast to the previous work where we observed a peak yield of close to 80% free charges, here the free-charge yield measured via time-resolved microwave conductivity (TRMC) is suppressed to <20%. We attribute this suppression to an ultrafast energy-transfer process that rapidly transfers donor excited states to the PCBM host at short-range (ca. <3 nm), ultimately resulting in localized CT state generation. Furthermore, calculated FRET rate constants (average *k*_*FRET*_ ca. 6 × 10^10^ s^–1^) for each donor with PCBM demonstrates that FRET alone does not account for necessary energy-transfer rate constant, from which we infer participation of an ultrafast process involving either Dexter energy transfer, or relaxation through an intermediate exciplex state (average *k*_*EET*_ ca. 1 × 10^12^ s^–1^) at far shorter ranges (ca. *<* 1 nm). Transient absorption (TA) and photoluminescence excitation spectroscopy (PLE) show that these transferred excited states do not diffuse away into the PCBM to decay via other pathways but undergo rapid hole transfer with the donor evinced by long-lived bleach signals and in some cases significant CT exciton emission. Finally, TRMC experiments conducted at excitation wavelengths exciting primarily PCBM at higher energy (565–640 nm) produced a free-charge yield curve as a function of Δ*G*_*CT*_ which is *not* suppressed as it is when exciting on the blue-sensitizer absorption peak (650–680 nm). These results show that the primary effect of the exciton energy transfer (EET) process is to confine charge-transfer events to the local sphere of PCBM molecules around the donor, precluding the possibility of long-range charge-transfer events that can produce free charges and biasing the system toward a preponderance of localized CT states. Indeed, we infer that the FC’s measured via TRMC when selectively exciting the blue sensitizers must primarily arise from that fraction of the light which is directly absorbed by the PCBM instead.

Figure 1a shows example absorption spectra of one of the blue sensitizers (remaining absorption and photoluminescence provided in the Supporting Information, SI Figures 1.1–1.3), SiPcBu, sensitized in both an inert polystyrene (PS) host matrix (gold) as well as the PCBM accepting host matrix (blue) compared to the neat PCBM host (black). We call these sensitizers “blue” because their peak absorption (ca. 650–680 nm) lies on the blue side of the PCBM absorption onset (ca. 730 nm), such that we can no longer *exclusively* excite the donor or preclude the possibility of energy transfer. Figure 1b demonstrates the spectral overlap between an example donor and the PCBM acceptor that is utilized in the calculation of *k*_*FRET*_. Due to the very small Stokes shift typical of all these sensitizers (ca. <2 nm for SiPcBu), and even though the absorption of neat PCBM is relatively weak in that region, the spectral overlap is sufficient to provide energy-transfer rate constants on the order of 1 × 10^10^ to 1 × 10^11^ s^–1^ (calculations for *k*_*FRET*_ and simulated *k*_*EET*_ provided in Table 2 and SI Figures 5.1 and 5.2).

**Figure 1:**
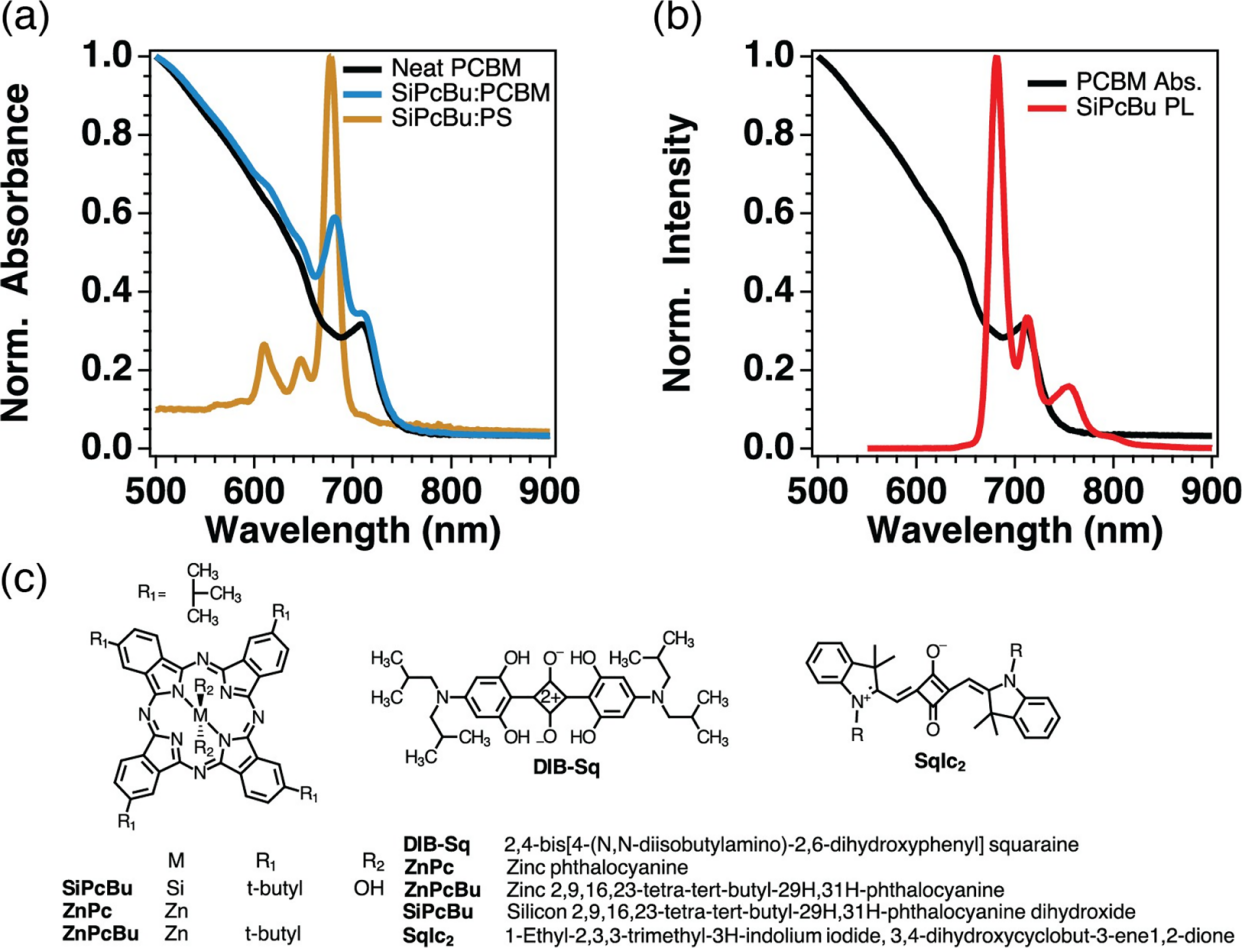
Molecular system of interest. (a) Normalized absorption spectra of a neat PCBM film (black) and a comparison of SiPcBu sensitized (0.005 mol kg^–1^) in an inert polystyrene host matrix (gold) and in the PCBM accepting host matrix (blue). (b) Normalized absorption of neat PCBM film overlapping with normalized PL of the SiPcBu:PS film demonstrating the spectral overlap of the donor emission and PCBM absorption. (c) Blue sensitizer molecular structures and abbreviations used in this work (see also Table 1).

Consistent with our previous work, we use absorption and emission spectroscopy to provide evidence of isolated sensitization of the donor into the PCBM accepting host.^12^ These data demonstrate spectral features that resemble molecular phthalocyanine and squaraine absorption, rather than typical aggregated features, such as dramatic red-shifting and/or broadening, which are absent here.^12,20−23^ However, all of the sensitizer absorption peaks do broaden and red-shift to varying degrees in the PCBM host relative to polystyrene. This could be simple solvatochromism in response to the higher dielectric constant of PCBM (ϵ_*r*_ = 4) vs polystyrene (ϵ_*r*_ = 2). However, it might also signal stronger electronic interactions between the PCBM and the sensitizer.^24,25^ This latter behavior was extensively investigated in the context of fullerene–porphyrin^26^ and fullerene–phthalocyanine^27−29^ dyads in solution, where very similar absorption shifts were correlated with the formation of an emissive long-lived exciplex, distinct from a charge-transfer state, and only slightly lower in energy than the local exciton of the phthalocyanine or porphyrin. The excitonic coupling was found to depend strongly on whether the fullerene was oriented face-on or edge-on relative to the plane of the macrocycle,^28^ which is consistent with the fact that Si-centered phthalocyanines and naphthalocyanines both in this study and our prior work^12^ tend to have spectra that are less perturbed than any of the others. These molecules all contain axial substituents on the Si atom (albeit only hydroxyl groups in some cases) that would be expected to disrupt close cofacial stacking and weaken the coupling. Finally, we note that, taking all the sensitizer molecules used both here and in our prior work^12^ together, there is no clear trend in the absorption shift as a function of the driving force for electron transfer. Rather, it appears more closely connected with the molecular structure: squarine derivatives show the largest spectral shifts, and Si-centered phthalocyanines and naphthalocyanines show the smallest. Given the relatively small difference in energy previously estimated between the molecular excited state and the exciplex^26^ in the more strongly coupled case of porphyrin–fullerene dyads, we do not believe that this effect significantly influences our methodology for calculating the driving force for electron transfer, particularly given that it is the lower PCBM exciton energy we use for the driving force calculation discussed below. Exciplex formation may nevertheless serve as a natural explanation for how energy transfer can be fast enough to out compete electron transfer in these systems; we return to this point later in the text.

Control over the photochemical driving force, Δ*G*_*PET*_, is achieved by choosing a series of blue sensitizers with varying oxidation potentials as measured by cyclic voltammetry (electrochemistry data is provided in SI Figure 2.1), which provides us with an approximately −0.5 eV range for Δ*G*_*PET*_ (see Table 1).

**Table 1:** Tabulated Average *E*_*ox,D*_, Δ*G*_*CT*_, and ϕ_*FC*_ for All Sensitizers in Both Primarily Sensitizer and Primarily PCBM Excitation Conditions^a^

Sensitizer	*E*_*ox,D*_^b^ (eV)	Δ*G*_*CT*_ (eV)	ϕ_*FC*_ Sens. exc.	ϕ_*FC*_ PCBM exc.
SiPcBu	0.46	–0.19 ± 0.04	0.07 ± 0.01	0.12 ± 0.01
DIB-Sq	0.37	–0.28 ± 0.03	0.10 ± 0.01	0.18 ± 0.01
ZnPc	0.25	–0.40 ± 0.02	0.18 ± 0.02	0.72 ± 0.01
ZnPcBu	0.12	–0.53 ± 0.01	0.11 ± 0.01	0.12 ± 0.01
SqIc2	0.05	–0.62 ± 0.01	0.05 ± 0.02	0.34 ± 0.01

a These data are from Figure 2. Δ*G*_*CT*_^12,14^ is calculated for each sensitizer:PCBM pair using measured *E*_*red,A*_ of −1.07 V for PCBM and the PCBM exciton energy, *E*_*ex*_ of 1.72 eV, since each of the blue sensitizers have excited-state energies higher than the PCBM excited state. All redox potentials are vs Fc/Fc^+^. ϕ_*FC*_ is calculated from TRMC assuming μ_*e*_ = 0.040 cm^2^ V^–1^ s^–1^. The error associated with Δ*G*_*CT*_ is propagated from the averaged oxidation and reduction potentials. The error associated with ϕ_*FC*_ is from differences in absorption across films as detailed in Experimental Methods. All CV scans are shown in SI Figure 2.1. The *E*_*ex*_ value for PCBM is estimated from absorption/PL spectra in SI Figure 1.4.

b Assuming a one-electron redox reaction, half-wave potentials can be expressed in units of eV.

The bulk of the experimental data is condensed into Figure 2, showing the photoinduced charge carrier yield (ϕ_*FC*_) measured by TRMC as a function of the Gibbs energy change for charge transfer to the most localized CT state (Δ*G*_*CT*_). Three data sets are displayed for comparison: (1) the blue-sensitizer system exciting primarily PCBM (565–640 nm, black squares); (2) the blue-sensitizer system exciting primarily the sensitizers (650–680 nm, blue squares); (3) the prior data from Carr et al.^12^ using red sensitizers, where the sensitizer is selectively excited (light red squares). The dashed curves are model results that will be discussed further below.

**Figure 2:**
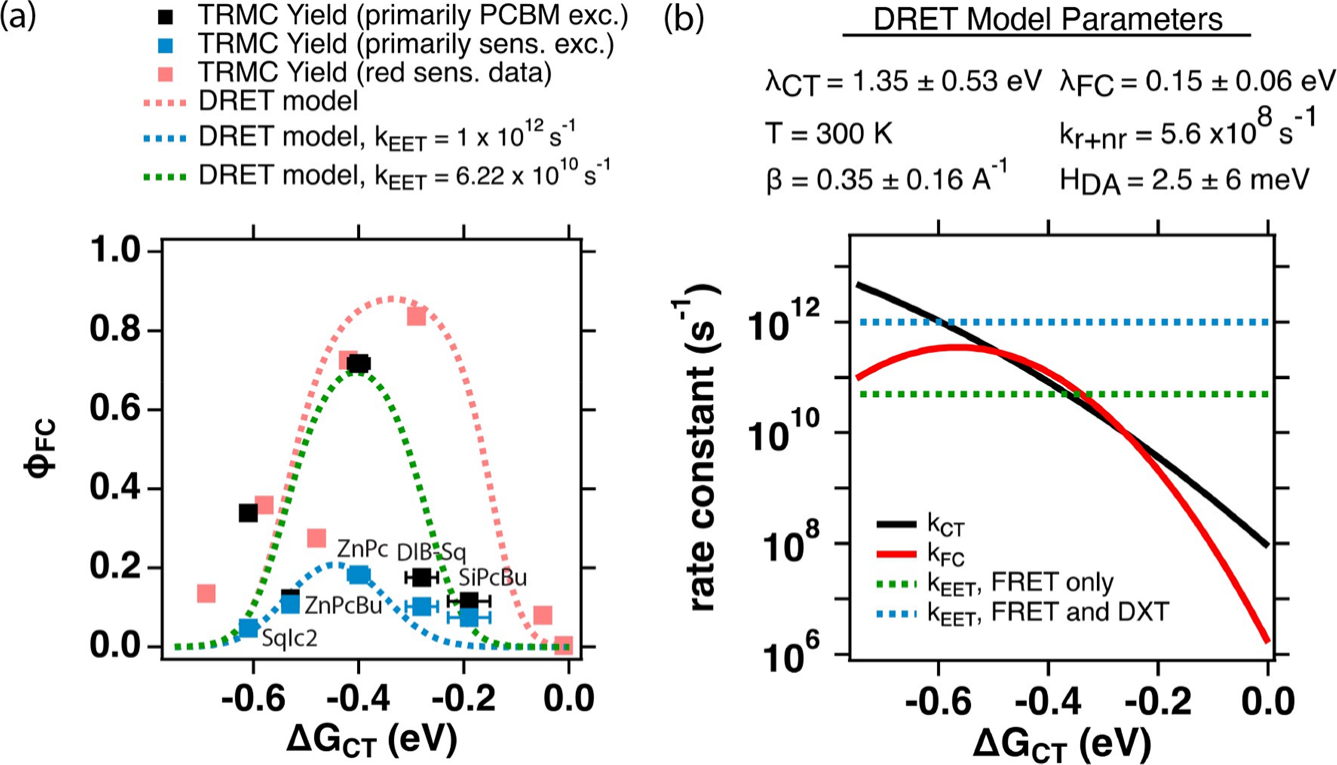
(a) Free-charge yield, ϕ_*FC*_, as a function of Δ*G*_*CT*_ as determined via TRMC (assuming μ_*e*_ = 0.040 cm^2^ V^–1^ s^–1^) for two different excitation conditions: (1) Exciting primarily the PCBM accepting host between 565 and 640 nm (black squares) and (2) exciting primarily the sensitizers between 650 and 680 nm (blue squares). The light red data points are the reproduced ϕ_*FC*_ from Carr et al.^12^ as a comparison. In addition there is also a comparison of ϕ_*FC*_ as predicted by the DRET model as a function of Δ*G*_*CT*_ for three different cases: (1, light red dashed trace) DRET model fit to ϕ_*FC*_ for the red sensitizer data, assuming that no energy transfer occurs in the system (assuming *k*_*EET*_ = 0), (2, green dashed trace) simulated ϕ_*FC*_ from the amended DRET model assuming that energy transfer occurs, but is only from a slower FRET process (assuming that *k*_*EET*_ = 6.22 × 10^10^ s^–1^), and (3, blue dashed trace) simulated ϕ_*FC*_ from the amended DRET model assuming that energy transfer occurs, and includes a faster Dexter process (assuming that *k*_*EET*_ = 1 × 10^12^ s^–1^). (b) PET rate constants, *k*_*CT*_ (black trace) and *k*_*FC*_ (red trace) as a function of Δ*G*_*CT*_ as calculated from the DRET model. The dashed lines are the constant values of *k*_*EET*_ as a function of Δ*G*_*CT*_ in the corresponding green and blue dashed lines in panel a. All simulations and calculations are evaluated using the fit parameters determined by fitting the ϕ_*FC*_ data from the red sensitizer data (light red squares and dashed trace) as was done in Carr et al.^12^ Errors provided in panel a are standard errors calculated from repeat measurements as described in Experimental Methods. Sensitizer Δ*G*_*CT*_ and ϕ_*FC*_ values with reported errors are in Table 1.

Two aspects of this experimental data presentation require some special explanation. First, the *y*-axis is labeled yield, when usually TRMC experiments can only provide the product between the mobile carrier yield and the sum of the electron and hole mobilities. In the present work, and similar to the experiments in Carr et al., we have designed our system with two features that allow us to quantify the yield of mobile electrons (ϕ_*FC*_) explicitly: (1) the hole mobility is zero due to the dilute concentration and isolation of electron donating sensitizers, and (2) the electron mobility of PCBM is known at our ca. 9 GHz microwave frequency from prior work from Warman et al. and Ferguson et al. (0.04–0.059 cm^2^ V^–1^ s^–1^).^30,31^

The second feature requiring special attention is our calculation of the Gibbs energy *x*-axis. Δ*G*_*CT*_ is given by a simplified version of the Gibbs free energy equation for photoinduced electron transfer:
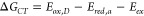
1where *E*_*ox,D*_ and *E*_*red,A*_ are the half-wave potentials of the donor and acceptor respectively, as measured via CV (values for each given in Table 1), and *E*_*ex*_ is the lowest-lying exciton energy in the system, which in this case is always the PCBM exciton energy (estimation from overlap of absorption and emission in SI Figure 1.4). The subscript (“CT”) in eq 1 denotes the physical meaning of the simplifications made to the full Gibbs energy change for PET. The full equation includes the electrostatic work^19^ and Born corrections,^32^ but it transpires that if one assumes the electron–hole distance in the exciton is equal to that in the nearest-neighbor CT state and that the dielectric constant of the solid is much smaller than that of the solution in which the electrochemical potentials were measured, then the Born and electrostatic work terms exactly cancel, leaving eq 1 to express the driving force *to that most localized state*.^12,14^ We employ this rather than attempting to calculate the Gibbs energy change with respect to free charges because Δ*G*_*CT*_ is based entirely on experimental data (CV and optical spectroscopy) whereas a calculation of Δ*G*_*FC*_ would necessarily involve adjustable parameters that are not precisely known.

Three things are immediately obvious in Figure 2a. First, all the data exhibits a clear optimum in carrier yield as a function of the Gibbs energy change for the reaction, qualitatively consistent with the idea that a Marcus rate expression^33,34^ contributes to the yield of free charges as we have observed in the past^15,35,36^ and as described in our distributed range electron-transfer model.^12^ Second, when the “blue sensitizers” are selectively excited, the yield of free carriers is dramatically suppressed relative to our prior work selectively exciting “red sensitizers” in the PCBM host. Third, exciting the “blue sensitizer” films at a wavelength that primarily excites the PCBM host recovers the peak yield, if not the exact shape of the curve obtained for the “red sensitizers”. The remainder of this work focuses on understanding these unexpected differences.

Here we make a brief digression to explain the principles behind the distributed range electron transfer (DRET) model that is used to fit the data in Figure 2a, and which is crucial to many of the logical arguments made throughout the remainder of the text. However, the treatment here is purely qualitative and the interested reader is referred to our prior work.^12^ DRET describes free-charge generation as a competition between two subsets of charge-transfer states: those far enough apart to escape their coulomb attraction that we refer to as free charge (FC) states and those that begin too close together that we refer to as charge-transfer (CT) states. The former is considered to be the productive “charge generation” channel, while the latter is modeled as a pure loss pathway. The dividing line between these species is chosen rather arbitrarily: it is the charge-transfer radius that is 1 kT below the Gibbs energy curve for full charge separation. The respective rate constants for these processes (and thus their yields) are found by using the classical Marcus equation (eq 2) and integrating the electron-transfer rate constant (*k*_*PET*_) over the available microstates as a function of charge separation radius.^5^ Within the integration, the electronic coupling term, *H*_*DA*_, is assumed to fall off exponentially with charge separation distance according to *H*_*DA*_e^–(*r*_*DA*_–*r*_0_)β^.^37^ In addition, the Gibbs energy change for electron transfer Δ*G* takes on a distance dependence due to the electrostatic work required to separate charges from an initial radius (*r*_0_) to a final radius (*r*_*DA*_). Finally, and crucially, we also allow the reorganization energy, λ, to be different for the FC (λ_*FC*_) and CT (λ_*CT*_) states, finding that the model only satisfactorily fits our data when the CT states have a much larger reorganization energy than the FC states.
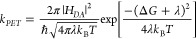
2

The parameters shown in the table at the top of Figure 2b are the result of fitting this model to the red-sensitizer data, and the main panel of Figure 2b shows the competing charge-transfer rate constants that result in red (*k*_*FC*_) and black (*k*_*CT*_). The final model result for free charge yield (ϕ_*FC*_) is shown as the dashed light red trace in Figure 2a. Thus, the “Marcus-like” optimum in the free charge yield we observe is actually the result of two overlapping Marcus curves, which leads to a certain asymmetry and a mismatch between where the peak of the curve lies and the reorganization energies in the model.

Returning to our experimental data, we first focus on the discrepancy in ϕ_*FC*_ between selective excitation of the red sensitizers and that of the blue sensitizers. Evidently there is a kinetic process that competes with free charge generation when the blue sensitizers are selectively excited, which is absent for the red sensitizers. The obvious candidate is some form of excitation energy transfer (EET) that ultimately precludes the production of free charges. This idea is summarized by eq 3, which describes the yield of free charges (ϕ_*FC*_) as a competition between the natural decay rate of the exciton (*k*_*r*+*nr*_), the rate constant for free charge generation (*k*_*FC*_), the rate constant for bound charge-transfer state generation (*k*_*CT*_), and that for excitation energy transfer (*k*_*EET*_).
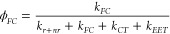
3

F̈orster resonance energy transfer (FRET) is often the dominant form of EET in molecular systems, but in this case, we are forced to conclude that a different process must dominate. We calculated the FRET rate constant for energy transfer from each donor molecule to the PCBM host based on its luminescence quantum yield, fluorescence lifetime, and spectral overlap between its emission and the PCBM absorption spectrum. Even after integrating over the quasi-infinite spherical shell of PCBM acceptor molecules surrounding an isolated sensitizer, on average, FRET is only 6 × 10^10^ s^–1^ due to the weak absorbance of PCBM in the relevant spectral region (FRET calculations and discussion in SI Section 5 and SI Figures 5.1 and 5.2). In contrast, the overall electron-transfer rate constant is an order of magnitude larger, which we infer from both TA experiments and fits using the DRET model.

The dashed curves in Figure 2a illustrate this point using the output of the DRET model for three cases: the first (light red dashed) is a fit of the original model to the red-sensitizer data (light red squares) where the free charge yield remains high and *k*_*EET*_ = 0. The second, intermediate case (green dashed), shows the result of simulating the DRET model when *k*_*EET*_ = ⟨*k*_*FRET*_⟩. The dashed green traces do not come close to fitting the blue-sensitizer data (blue squares). Finally, the last case is the result of simulating the DRET model with much larger generalized EET rate constat: *k*_*EET*_ = 10^12^ s^–1^ (blue dashed curve).

Interestingly, this simple introduction of a competing EET rate constant also has the effect of accounting for the shift of the optimal free charge yield toward more negative Gibbs energy values. Figure 2b shows why. The free charge yield is determined by the competition between *k*_*EET*_ (blue or green), *k*_*CT*_ (black), and *k*_*FC*_ (red). Thus, as *k*_*EET*_ shifts to larger values, only the larger charge-transfer rate constants can compete. Table 2 summarizes these results numerically, including the experimentally determined *k*_*FRET*_ for each sensitizer, the values of *k*_*FC*_ and *k*_*CT*_ predicted by the DRET model fit to the red-sensitizer data, and the value of overall energy-transfer rate constant (*k*_*EET*_) needed to approximately fit the blue-sensitizer data. We thus infer that there must be an alternative energy-transfer pathway with a rate constant approaching 10^12^ s^–1^ in every case. One candidate for this is Dexter energy transfer,^38^ while another mechanism could be mediated by relaxation of an intermediate exciplex, discussed above. The present data do not allow us to distinguish between these processes, and we simply note that both of them are consistent with the moderately strong electronic interaction between the sensitizer and the fullerene host that may cause the previously discussed shifts in the sensitizer absorption spectra.

**Table 2:** Rate Constant Values for Comparison of *k*_*FRET*_, *k*_*FC*_, *k*_*CT*_, and *k*_*EET*_^a^

Sensitizer	*k*_*FRET*_ (s^–1^)	*k*_*FC*_ (s^–1^)	*k*_*CT*_ (s^–1^)	*k*_*EET*_ (s^–1^)
SiPcBu	2.09 × 10^11^	1.84 × 10^9^	2.67 × 10^9^	1 × 10^12^
DIB-Sq	5.33 × 10^10^	1.70 × 10^10^	1.18 × 10^10^	1 × 10^12^
ZnPc	1.34 × 10^10^	1.15 × 10^11^	7.15 × 10^10^	1 × 10^12^
ZnPcBu	2.33 × 10^10^	2.32 × 10^11^	3.98 × 10^11^	1 × 10^12^
SqIc2	1.07 × 10^11^	1.78 × 10^11^	1.02 × 10^12^	1 × 10^12^

a *k*_*FC*_ and *k*_*CT*_ values are calculated using the DRET model (eq 3) integrated over all possible microstates as is done in Carr et al.^12^*k*_*FRET*_ is determined through a series of experiments and calculations detailed in SI Section 5. *k*_*EET*_ is held constant at a lower limit necessary to account for the discrepancy in ϕ_*FC*_ from Figure 2.

There are two possibilities that could explain why free charge generation is suppressed by this energy-transfer process: (1) the transferred exciton simply diffuses away with high probability and never participates in charge transfer, or (2) the transferred exciton still participates in charge transfer but only forms bound charge transfer states that are not detectable by our microwave conductivity experiment. The latter turns out to be the case, as shown by a combination of TRMC experiments directly exciting the PCBM, photoluminescence excitation spectra, and transient absorption measurements.

The microwave conductivity data for direct excitation of the PCBM is shown by the black squares in Figure 2a and has already been presented briefly. Here, emission wavelengths between 565 and 640 nm were chosen depending on the sensitizer so as to primarily excite the PCBM, and do so with an optical density that is as near as possible equivalent to that achieved for selective excitation of the corresponding sensitizer. This keeps the realized excitation density equal, allowing direct comparisons. Evidently, excitons that are injected directly into the PCBM efficiently diffuse to the available sensitizer molecules and participate in the charge transfer. Indeed, their free-charge yield is far higher than when the sensitizers are excited directly (blue squares) *for the exact same samples*. Thus, there is no reason to expect that an exciton *transferred* to the PCBM would fail to engage in electron transfer, a result that is further corroborated by the photoluminescence excitation and transient absorption measurements described below. The one case where we do not see any increase in free-charge yield for excitations into PCBM is in the ZnPcBu:PCBM sample (see SI Figure 3.5 for details). This particular sample is observed to retain a low free-charge yield mostly independent of excitation wavelength. We believe that this sample might be affected by other systematic variables that we cannot account for. If, for example, the ZnPcBu molecules are distributed less evenly throughout the film than the other sensitizers, this would tend to inhibit PCBM exciton dissociation, as the encounter probability could be lower. We stress however that only one out of the five samples fails to show a significant increase in free-charge yield when the PCBM is selectively excited.

A series of photoluminescence quenching (PLQ) and photoluminescence excitation (PLE) experiments summarized in Figure 3 provide key evidence for the ultimate fate of transferred excitons in this system, showing the results for the sample which produces the most free charges in the “blue sensitizer” series: ZnPc:PCBM. First, it is clear from Figure 3a that the molecular luminescence of ZnPc is strongly quenched, as anticipated. We estimate the photoluminescence quenching by analyzing the difference between the overlapping spectra of the ZnPc:PS film with the ZnPc:PCBM film at 730 nm and find quenching efficiency of *>*90%. Applying similar methodology to the whole series of donors in this study shows the same result, except for SiPcBu, which also exhibits the lowest driving force for electron transfer (see SI Figure 1.5 and SI Table 1.1). Figure 3a also demonstrates that there is a qualitative change in what emission remains in the ZnPc:PCBM sample, matching neither the ZnPc nor the PCBM spectra. A broad red-shifted emission feature appears, centered at 900 nm, that we attribute to CT state emission in this sample. The inset plot shows this difference clearly, denoting the two regions where we detect our PLE spectra (730 and 900 nm), shown in Figure 3b,c. Qualitatively altered emission spectra are observed for all of the sensitized PCBM samples except SqIc2, where the only detectable emission matches that of PCBM.

**Figure 3:**
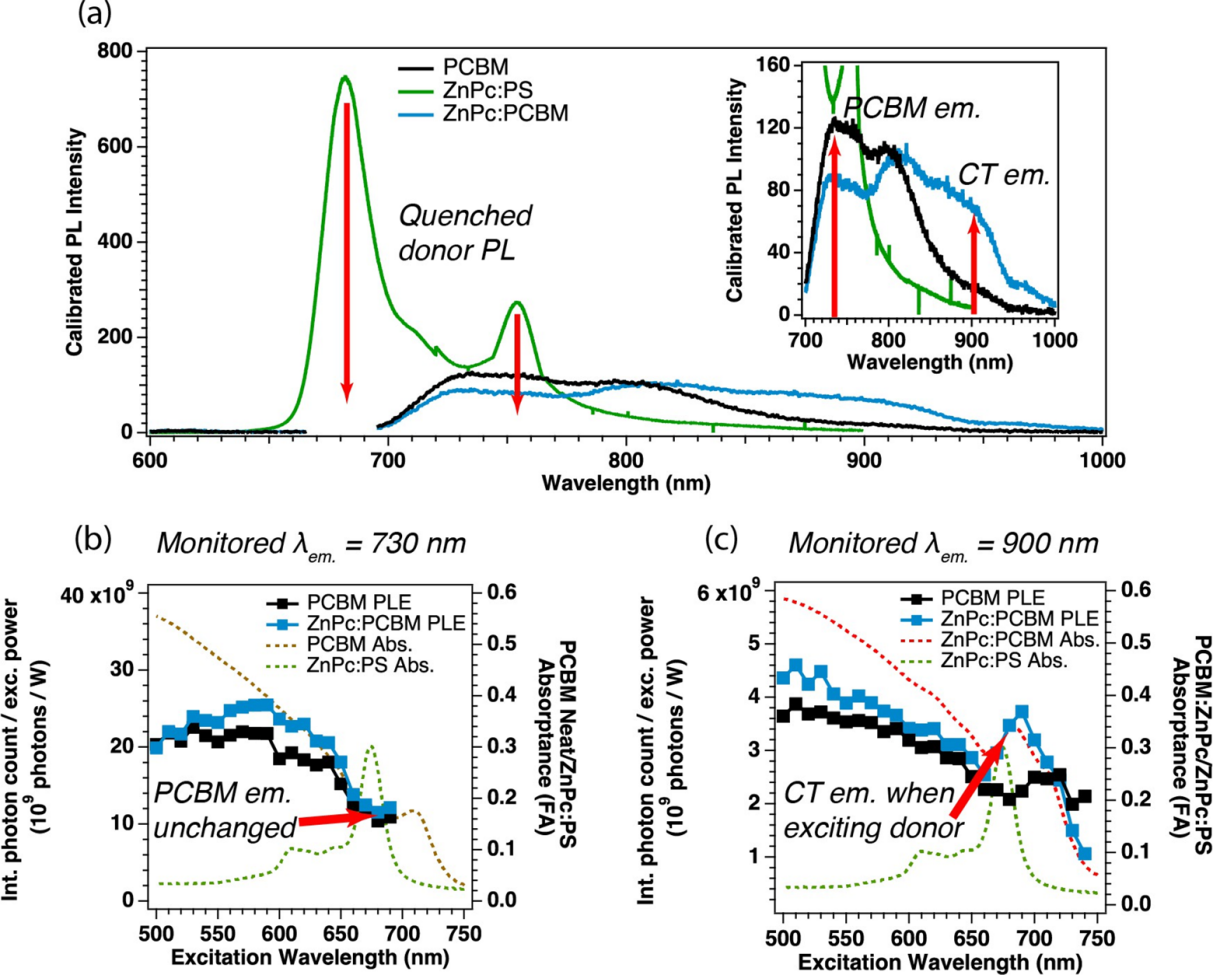
(a) Photoluminescence spectra of neat PCBM (black trace) and ZnPc:PCBM (blue trace) excited at 680 nm at the peak of the sensitizer absorption, where TRMC and TA experiments are conducted, compared to ZnPc:PS (green trace) excited at 380 nm to keep excitation and emission features well separated. Remaining excitation light (665–695 nm) is removed from the PCBM film spectra for clarity. The red arrows drawn from the ZnPc:PS trace down to the baseline are demonstrating the quenching of ZnPc emission features in the PCBM host films. The peak at 680 nm is some excitation light making it to the detector, but a 700 nm long-pass filter is used to reduce 680 nm excitation density in the spectrum. The inset plot is shown to enhance the resolution on the intensity axis to show the difference in the neat PCBM and ZnPc:PCBM films. The red arrows on the inset plot denote monitored center wavelengths for the photoluminescence excitation experiments at the peak of the PCBM emission at 730 nm and at the center of the broad CT emission feature at 900 nm. (b and c) Excitation wavelength, power-corrected, photoluminescence excitation spectra of neat PCBM (black squares and trace) and ZnPc:PCBM (blue squares and trace) films monitored at center wavelengths of 730 nm (b) and 900 nm (c). In both spectra, absorption of the ZnPc:PS film (b and c, green dashed trace) and neat PCBM absorption (b, brown dashed trace) or ZnPc:PCBM (c, red dashed trace) are given as comparisons to the PLE spectra.

PLE experiments provide the absorptance spectrum of the species that is complementary to emission at the wavelength chosen. This makes them ideal for identifying energy or charge-transfer processes, as the absorptance profile of an energy donor will appear contributing to the emission of the energy acceptor. Figure 3b,c shows the PLE spectra for neat PCBM and ZnPc:PCBM films monitored at 730 nm (Figure 3b) and 900 nm (Figure 3c). The emission feature at 730 nm, which is attributed to the peak of the PCBM emission spectrum, remains qualitatively unchanged in the presence of the ZnPc sensitizer and matches well with the neat PCBM absorptance (brown dashed line). However, the emission feature at 900 nm, which is attributed to the center of the CT emission band, is qualitatively different in the presence of the sensitizer. The ZnPc absorptance spectrum is clearly seen contributing to (presumed) CT emission band at 900 nm, whereas it does not contribute to characteristic PCBM emission at 730 nm.

Transient absorption spectroscopy also demonstrates that transferred excitons do not just diffuse away but undergo ultrafast hole transfer back to the sensitizer. Transient absorption data of the blue sensitizers dispersed in a PS matrix (SI Figure 7.1) exhibit behavior consistent with excited-state decay dependent on the molecular structure; that is, the squaraines behave similarly and the phthalocyanines match each other. The principal difference is that all the phthalocyanines exhibit efficient intersystem crossing with approximately 50% triplet yield, whereas the squaraines undergo simple decay of *S*_1_ back to the ground state. A detailed description of the TA spectra is available in the Supporting Information. In the PCBM host (SI Figure 7.2), the exhibited behavior is dependent on the driving force with a clear distinction between spectra on the lower end and higher ends of the driving force curve. Figure 4 summarizes the differences in behavior between the sensitizers in PS and PCBM across the driving force curve at photoexcitation wavelengths consistent with a majority of excitations being absorbed by the sensitizer. Table 3 displays the fits to the data using either a single or biexponential decay function with an offset (*y*_0_) indicative of how much signal remains at the end of our observation window (5 ns). At the lowest driving force, SiPcBu:PCBM (Figure 4a) shows a rapid quenching of the GSB compared to that of the SiPcBu:PS case. This fast component accounts for approximately 42% of the decay, with a time constant of 9.6 ps (Table 3). The other 40% of the decay matches the kinetics of the SiPcBu:PS case with ca. 15% of the excited states persisting beyond 5 ns in both samples.

**Figure 4:**
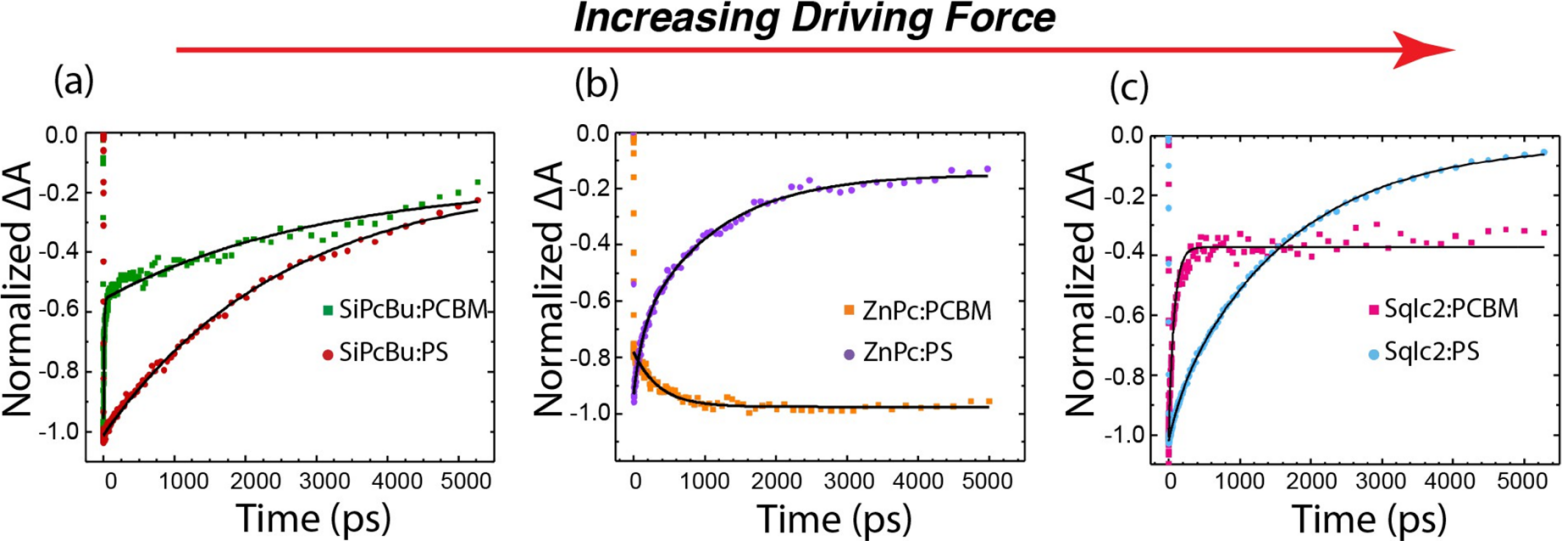
Transient absorption kinetics monitored at 680 nm and fits (black lines), the ground-state bleach (GSB) of the blue sensitizers in either a PS or PCBM host at three different driving forces, increasing from left to right: the lowest driving force: (a) SiPcBu:PCBM (green) and SiPcBu:PS (red) excited at 700 nm (100 nJ/pulse); the optimal driving force: (b) ZnPc:PCBM (orange) and ZnPc:PS (purple) excited at 700 nm (100 nJ/pulse); and the highest driving force: (c) SqIc2:PCBM (pink) and SqIc2:PS (light blue) were excited at 650 nm (100 nJ/pulse). Results of the fits are shown in Table 3.

**Table 3:** Results of TA Kinetic Exponential Fits at 680 nm for SiPcBu, ZnPc, and SqIc2 Dispersed in Both Polystyrene and PCBM Hosts

Sample	*y*_0_	*A*_0_	τ_0_ (ps)	*A*_1_	τ_1_ (ps)
SiPcBu:PS	–0.15 ± 0.001	–0.85 ± 0.001	2520 ± 64	–	–
SiPcBu:PCBM	–0.16 ± 0.04	–0.42 ± 0.01	9.6 ± 0.6	–0.4 ± 0.04	3042 ± 557
ZnPc:PS	–0.15 ± 0.007	–0.21 ± 0.01	116 ± 13	–0.57 ± 0.01	1096 ± 57
ZnPc:PCBM	–0.98 ± 0.002	0.2 ± 0.003	355 ± 15	–	–
SqIc2:PS	–0.03 ± 0.004	–0.1 ± 0.006	175 ± 14	–0.89 ± 0.005	1656 ± 28
SqIc2:PCBM	–0.37 ± 0.004	–0.63 ± 0.005	75.6 ± 2.1	–	–

This is consistent with the steady-state PL quenching experiments, which show 52% quenching efficiency for this sensitizer in PCBM (see Table S1.1). However, the TA spectrum is distinctly different for SiPcBu:PCBM, as is its emission spectrum (see Figure S1.5) compared to SiPcBu:PS. These results suggest that the dominant deactivation pathway at the lowest driving force is energy transfer from the donor to acceptor, consistent with other observations made throughout this Letter.

The nature of the long-lived spectrum and the emission is less clear but might be explained by the exciplex state suggested in our discussion of the absorption spectra, above. Contributions from the PCBM triplet and/or long-lived charge-transfer states are possible but would not readily explain the strong residual emission.

Figure 4b represents the optimal driving force case of ZnPc:PCBM. Contrasting SiPcBu:PCBM, the GSB of ZnPc in the PCBM host film persists throughout the 5 ns window of this TA experiment, while ZnPc:PS exhibits the aforementioned excited-state decay processes. A small growth of approximately 20% in the GSB of the ZnPc in PCBM is observed with a time constant of 350 ps. To investigate the origin of this growth, we conducted a TA experiment selectively exciting the PCBM host at 600 nm (SI Figure 7.3). In this case, the GSB of the sensitizer undergoes a large multiexponential growth throughout the 5 ns window, consistent with diffusion of a PCBM excited state to a ZnPc molecule, followed by charge separation. Based on this evidence, we assign the 350 ps growth when exciting primarily the ZnPc at 700 nm to the small number of excitations that originate in the PCBM undergoing charge separation with the ZnPc. This, combined with the TRMC results for direct excitation of the PCBM showing high free charge yields, suggests that most if not *all* of the free charges we observe via TRMC when we attempt to selectively excite the blue sensitizers arises from the minority of the light that is instead absorbed directly by the PCBM.

At the highest driving force, SqIc2:PCBM (Figure 4c), the GSB of SqIc2 in the PCBM host undergoes a rapid decay of 76 ps, followed by a persistent signal that lasts beyond 5 ns. In this case, the PCBM absorption centered at 550 nm and the GSB at 680 nm have the same kinetics (see SI Figure 7.4 for the spectra of neat PCBM). There is minimal evidence of contributions of the SqIc2 PIAs from the excited state in the TA spectra, so the kinetics at 550 nm should not be affected by SqIc2 contributions. In fact, the SqIc2:PCBM and ZnPcBu:PCBM TA spectra (SI Figure 7.2D,E, respectively) look qualitatively similar compared with the rest of the series. As these are both on the higher end of the driving force curve and exhibit similar kinetic profiles, we assign this behavior to rapid formation of CT states.

The initial fast decay kinetics of the GSB for all samples *except* ZnPc are consistent with two possible explanations: EET proceeds without significant ET subsequently to generate CT states, or a significant fraction of those CT states are short-lived and control the kinetics observed. The spectral shapes help to distinguish the competing hypotheses. In all cases, the transient spectra of the sensitizer:PCBM samples cannot be described as simply a linear combination of sensitizer and PCBM ESA features, showing clearly that the formation of CT states is occurring, even at the earliest recorded time delays (see the Supporting Information for control experiments and details). In all cases but SiPcBu (the lowest driving force sample) the long-lived component of the GSB kinetics is significantly modified across the sensitizer series. We propose that this difference as a function of driving force is most consistent with a distribution of CT states whose recombination rate constant controls the GSB decay in each sample, encompassing both the slow and fast components of the decay. The larger ratio of the longer-lived species in the ZnPc is the reason we can observe a slow rise compared to the other samples. The slow rise coming from the fraction of excitations into the PCBM which is diffusion controlled would *only* be visible in samples where the competing decay process is slow enough to reveal it, as is the case in the ZnPc sample.

Figure 5 illustrates the proposed kinetic schemes and spatial cartoons that incorporate the conclusions drawn from the spectroscopic data above. Panels a and b depict the events that occur for direct excitation of a “blue sensitizer” in PCBM: a fast EET process competes with the initial PET step and *significantly reduces* FC yields. After EET, hole transfer back to the sensitizer proceeds efficiently but has the effect of forcing formation of a bound nearest-neighbor CT state. Panels c and d show the contrasting situation of direct excitation of the PCBM. Here, exciton diffusion efficiently brings excitons within range of the sensitizers for charge transfer and effectively samples the range of charge-transfer distances needed to produce free charges. These kinetic schemes are consistent with the DRET model prediction that the nearest-neighbor CT states in this system do not *mediate* free-charge generation but instead are a loss pathway, as otherwise one would expect that fast EET processes would only increase your charge separation efficiency. In Supporting Information Sections S8 and S9 we discuss, and ultimately refute, two competing hypotheses concerning how recombination to triplet states might quench the apparent free charge yield and the possible involvement of delocalized PCBM excitons in enhancing the free charge yield when PCBM is directly excited.

**Figure 5:**
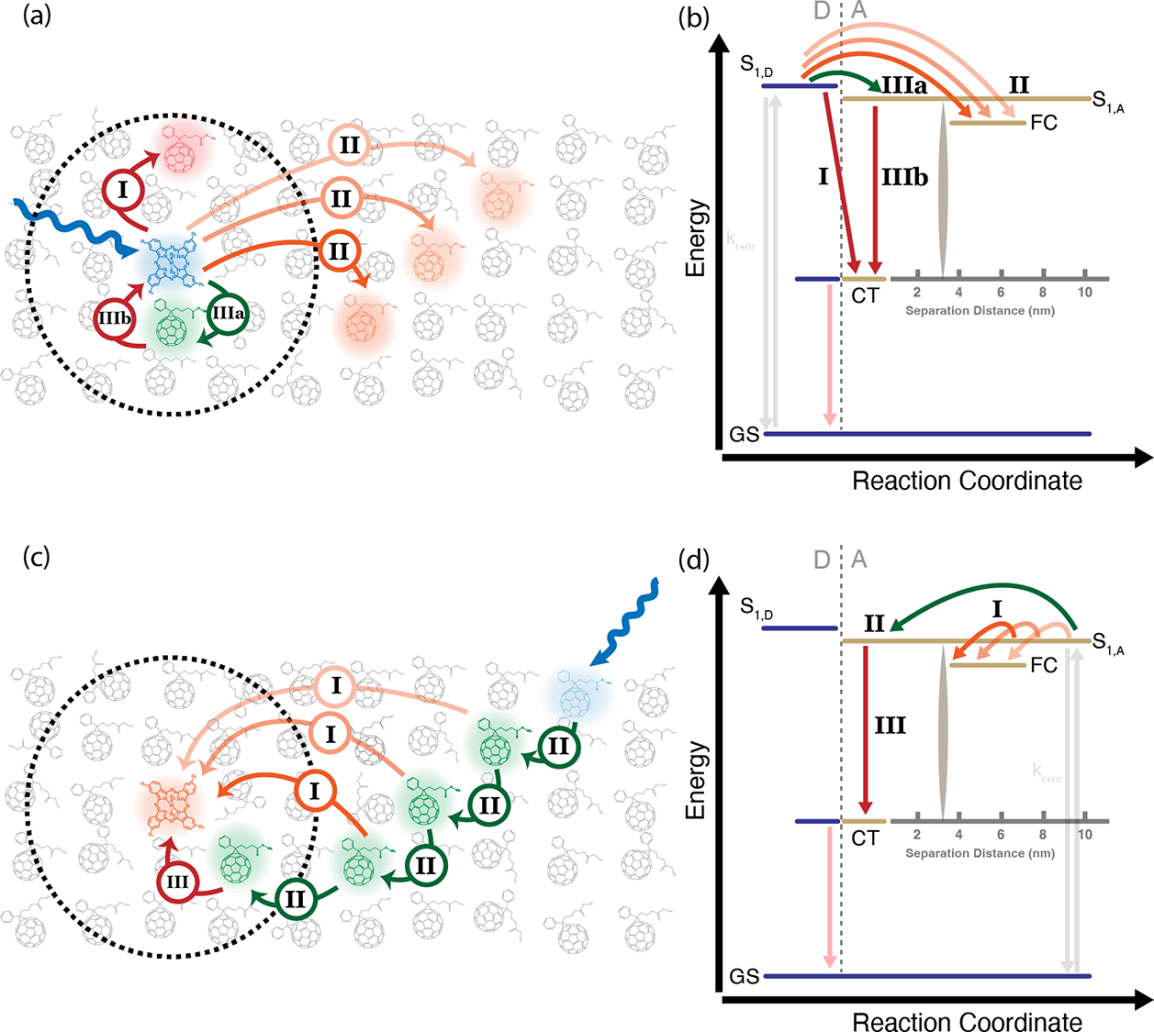
Spatial illustrations and corresponding kinetic schemes of the molecular system demonstrating two different scenarios possible from excitations into the film: (1) Excitations into the sensitizer (a and b) and (2) excitations into the PCBM host (c and d). In both cases, blue arrows refer to the excited molecule of interest, green arrows denote energy transfer (a) and diffusion processes (c), orange arrows denote PET processes that lead to free charges, and red arrows denote PET processes that lead to charge-transfer states. The dashed black circle is the critical radius at which we delineate distances where free charges are generated. (b) Proposed kinetic model for excitations into the sensitizer which demonstrate three competing processes from the excited donor singlet state, *S*_1*,D*_: (I) short-range CT state generation from the donor to PCBM; (II) long-range FC state generation at a variety of distances from the donor to PCBM; (IIIa) fast, short-range excitation energy transfer from the donor to PCBM; followed by (IIIb) CT state generation from PCBM to the donor. (d) Proposed kinetic model for excitations into the PCBM host which demonstrates two competing processes from the excited acceptor singlet state, *S*_1*,A*_: (I) long-range FC state generation at a variety of distances from the PCBM to the donor, (II) exciton diffusion to a variety of distances from which either FC or CT state generation can occur, and (III) short-range CT state generation from the PCBM to the donor. In both schemes, bimolecular recombination occurs from the FC states through the CT state, and the excitons and CT states may decay back to the ground state.

The experiments outlined above serve as an unexpectedly powerful test of our previously reported distributed range electron transfer (DRET) model: localizing the charge-transfer event to the nearest-neighbor shell of acceptor molecules via an energy-transfer channel dramatically reduces the free-charge yield. When direct excitation of the PCBM restores the ability of the system to explore the full range of possible electron-transfer events (including long-range events), the optimal free charge yield returns to its original value. Using a fixed set of parameters from fitting the red-sensitizer data from prior work, the DRET model accurately describes the optimal position of free-charge yield in the blue-sensitizer system, tracking a shift to higher driving force, with the only free parameter being an average energy-transfer rate constant that competes with free charge generation.

However, these results are also quite surprising in the context of existing literature. We find that the most localized CT states in our dilute donor:PCBM system are a loss pathway, not the key intermediate to free charge that is so often proposed in the operation of closely related organic photovoltaic materials.^39^ It has been shown, for instance, that in many organic photovoltaic systems the external quantum efficiency in devices remains near 100% even for the lowest-energy charge-transfer states that are detectable via highly sensitive photocurrent measurements.^40^

In reconciling this apparent conflict, a first important point is that DRET *also* predicts a significant free charge yield from subgap excitation of the CT manifold in bulk heterojunction blends.^12^ In our model this arises from direct excitation of longer range charge-transfer states and is largely driven by the fact that a blend system has a much larger entropic contribution to charge separation: there are both fewer nearest-neighbor states and more long-range ones at a planar interface than in a dilute donor–acceptor system.^5^ This already brings our results into partial agreement with the literature cited above. We are not the first to suggest the crucial importance of longer-range charge-transfer events, both from an experimental^11^ and theoretical perspective.^7^ A second important point is that excitation energy-independent photocurrent generation from CT states is by no means a universally observed property of organic photovoltaic devices.^41^

Nevertheless, the agreement noted above is only partial, as there *is* much experimental data in the literature consistent with efficient photocurrent generation arising from what are interpreted to be nearest-neighbor charge-transfer states.^40,42^ There are many models for how CT-state dissociation can occur spontaneously and efficiently, invoking disorder,^5,41,43,44^ delocalization,^2,3,45−47^ tunneling,^48^ and micro electrostatic fields.^8,49^ Our purpose in this paper is not to refute prior work but to present a different mechanism of charge separation that may operate cooperatively with those noted above to enable the shockingly efficient organic photovoltaic devices now prevalent in the literature.^50^ The charge generation behavior of the model systems we study in this paper and our prior work^12^ cannot be explained through spontaneous dissociation of nearest-neighbor CT states, yet they can produce free charge with high efficiency under the right circumstances. In showing that spontaneous CT state dissociation is not a necessary requirement for efficient free charge generation, we also raise the question of whether it is *desirable*. Disorder, for instance, has been shown to be a viable pathway to CT state dissociation but has other deleterious consequences.^3,41^ Our distributed-range electron-transfer model offers a new way of thinking about the driving-force dependence of free charge generation in OPV systems, which could prove to be an extremely useful tool in understanding how to optimize the open-circuit voltage in state-of-the-art devices.

In summary, we have discovered that in a dilute-donor/acceptor blend (phthalocyanine and squaraine donors in a PCBM acceptor matrix) the exciton energy of the donor can have a vital impact on whether free charge is produced efficiently or not and that this goes beyond the obvious consideration of how exciton energy controls the Gibbs energy change for photoinduced electron transfer. When the dilute-donor (sensitizer) is selectively excited, the free charge yield is dramatically suppressed when its exciton energy is above that of the host material, relative to what is observed when the reverse is true. We attribute this behavior to an ultrafast excitation energy-transfer mechanism followed sequentially by a hole transfer back to the sensitizer. This sequence of events forces charge transfer to occur in a very short range, leading primarily to the production of localized charge-transfer states that are not detectable by microwave conductivity. We infer that the EET step must proceed primarily via a Dexter or exciplex mediated mechanism, as calculations of the F̈orster rate constant show it to be 10× too small to explain our observations.

Photoluminescence excitation and transient absorption spectra show that quenching of the sensitizer emission is still quite efficient (*>*90% for all but one) for the blue sensitizers, confirming that the fate of the transferred exciton is to become trapped at the charge-separation interface, doomed to become a bound CT state. These results are consistent with our previously described distributed-range electron transfer (DRET) model, which describes free charge generation as a competition between short- and long-range charge-transfer events to localized charge-transfer (CT) states and delocalized free charge (FC) states, respectively. The results reported herein cannot be reconciled with a model that posits nearest-neighbor charge-transfer states as the *intermediate* in the production of the free charges detected by our microwave conductivity experiments. We propose that the mechanism described by DRET operates in parallel with those previously introduced to understand systems that *do* exhibit efficient CT state dissociation. In mapping out the role of the photochemical driving force on charge separation, DRET could be a powerful tool in understanding what the limits and optimum design strategies are for maximizing the open-circuit voltage of state-of-the-art devices.

Notably, these conclusions should not be construed as the repudiation of energy transfer as a useful mechanism to enhance the performance of organic photovoltaics. The reduction in the free charge yield we observe here arises because of an asymmetry in the entropy associated with charge transfer in each direction in a dilute blend. In bulk heterojunction structures, no systematic asymmetry is expected to exist, and free charge generation can be expected to proceed efficiently subsequent to excitation energy-transfer events.

## Experimental Methods

### Film Fabrication

Phenyl C_61_ butyric acid methyl ester (PCBM) was acquired from Nano-C with 99.9% purity. Zinc 2,9,16,23-tetra-*tert*-butyl-29H,31H-phthalocyanine (ZnPcBu), 2,4-Bis[4-(N,N-diisobutylamino)-2,6-dihydroxyphenyl] squaraine (DIB-Sq), and zinc phthalocyanine (ZnPc) were acquired from Sigma-Aldrich at *>*96% purity. Silicon 2,9,16,23-tetra-*tert*-butyl-29H,31H-phthalocyanine dihydroxide (SiPcBu) was acquired from American Elements at 99% purity. 1-Ethyl-2,3,3-trimethyl-3H-indolium iodide, 3,4-dihydroxycyclobut-3-ene1,2-dione (SqIc2) was synthesized according to Barbero et al.^51^ All molecules were used as received.

Sample films were fabricated by ultrasonic spray-coating host-sensitizer solutions onto 25 × 11 mm^2^ quartz substrates cleaned with acetone sonication for 10 min and 10 min of UV-ozone treatment. Stock solutions were prepared by dissolving each sensitizer in chlorobenzene at 1 mg/mL, except for ZnPc which was dissolved in pyridine at 1 mg/mL. PCBM and PS solutions were dissolved in chlorobenzene at 30 mg/mL. Host-sensitizer solution mixtures were made by mixing sensitizer solution with PCBM or PS host solution at 0.005 mol kg^–1^ for a total volume of 1 mL. All films were spray coated in a nitrogen glovebox (*<*1 ppm of O_2_). Spraying was accomplished by rastering the sample stage beneath the ultrasonic spray nozzle to coat a 50 × 60 mm^2^ area containing three 25 × 11 mm^2^ quartz substrates for making samples in triplicate under the same conditions. Atomized solution was delivered to the sample at a rate of 0.4 mL/min using a syringe pump, and air-shaping was applied with a 6 L/min nitrogen stream to achieve fan-like jets for uniform spraying. The sample stage was heated to 100 °C to facilitate evaporation of the high boiling solvents. Nozzle to substrate height was ca. 50 mm. Five coats (repetitions of the raster routine) were done to achieve films ca. 1 μm in thickness. PS and PCBM host films are made from the same spray coating parameters.

### Absorption Measurements

Optical absorption is characterized using a Varian Cary 5000 UV–visible spectrophotometer with the diffuse reflectance accessory (DRA) and an angled center mount. Spectra are collected in the transmittance configuration, but because we collect with the center mount in the DRA, it is effectively a transreflectance (%TR) spectrum, as both the reflectance (%R) and transmittance (%T) are collected simultaneously. Excitation of the sample is with the full beam size, which is centered on the film at an angle of incidence at 20°. The resolution of the instrument is 1 nm with grating changeovers at 800 and 350 nm. A baseline is collected by inserting a blank, cleaned quartz substrate into the center mount of the DRA under the same collection settings. Both a 100% transreflectance and a 0% transflectance, where the beam is blocked, are collected to baseline the instrument before collection. Absorptance (%A) is then calculated from the resulting spectrum by %A = 100% – %TR.

### Photoluminescence Spectroscopy

Photoluminescence spectra were collected using a custom-built Princeton Instruments spectrometer. A liquid-nitrogen-cooled, front-illuminated Si CCD (PyLoN) was used for collecting visible-NIR spectra (425–900 nm), and a 1D liquid-nitrogen-cooled InGaAs array (PyLoN-IR) was used for SWIR measurements (850–1550 nm). Vis-NIR spectra were intensity calibrated by using an IntelliCal USB-LSVN (9000–410) calibration lamp. SWIR spectra were calibrated using a SWIR quartz tungsten halogen lamp from Princeton Instruments. Dual monochromators (HRS 500) were used to achieve pseudomonochromatic excitation from an Energetiq EQ99x laser-driven light source, with typical fwhm bandwidths ca. 16 nm using a 1200 g mm^–1^, 750 nm blaze grating. A single monochromator was used for detection (Princeton HRS-300) with 1200 g mm^–1^ (500 nm blaze) and 150 g mm^–1^ (800 nm blaze) gratings used for measuring vis-NIR and SWIR spectra, respectively. Typical exposures were 0.5–1 s with 0.25–1 mm detection slit widths. PL spectra for each sensitizer:PS film were excited between 350 and 400 nm. Further information about photoluminescence measurements, including photoluminescence quenching and quantum yield experiments, can be found in the SI.

### Electrochemistry

CV measurements were done in triplicate for each sensitizer and the PCBM against the Fc/Fc+ standard reference in an inert glovebox environment (*<*1 ppm of O_2_). Experiments were performed on solutions of the sensitizer and PCBM in a 4:1 v/v ratio of dichlorobenzene to acetonitrile (Sigma-Aldrich 99.9% anhydrous grade) with 0.1 M *NBu*4^+^PF_6_^–^ (Sigma-Aldrich *>*99% electrochemical grade) in order to make sure that both the electrolyte and the analyte were dissolved entirely. Electrochemistry Power Suite software was used to control equipment and execute scans. Three cyclic scans were done prior to each cyclic voltammogram collection to ensure analyte equilibration with electrode surfaces. Scan rates varied from 100 to 200 mV/s, and each solution was scanned in both directions to ensure symmetry and reversibility. A “compact voltammetry cell research kit” (Pine product # AKSPEKIT) was used to ensure the best repeatability of electrode placement from sample to sample. The cell includes a screen-printed three-electrode system with a 2 mm Pt working electrode, a Pt counter electrode, and a silver wire pseudoreference electrode. The electrodes and silver wire are rinsed and sanded between each measurement to prevent any contamination. Following scans, the *E*_1*/*2_ of the first oxidation potential for the sensitizer is used to approximate the energy level of the donor and the *E*_1*/*2_ of the first reduction for the PCBM is used to approximate the energy level of the acceptor, both with reference to the Fc/Fc^+^ standard *E*_1*/*2_. This procedure is inspired by work from Larson et al.^52^ and has been used successfully in other prior work in Carr et al.^12^

### Time-Resolved Microwave Conductivity

The TRMC technique has been described in detail in previous publications both in terms of the theory and the experimental setup.^53,54^ Film photoconductivity for this work is determined by the following: (1) TRMC transients are collected as a function of light intensity for each sample in the series to ensure that the response is linearly correlated. (2) Transients are fit with biexponential functions convoluted with the 7 ns cavity response. (3) The resulting peak value is normalized by the fraction of absorbed photons in the film. A Spectra-Physics PremiScan ULD/500 optical parametric oscillator pumped by a Spectra-Physics Quanta-Ray Nd:YAG laser was used to excite the samples with ca. 7 ns pulses in the peak absorption for each sensitizer as shown in the inset on the absorption figures in the SI Figure 1.1. TRMC transients with fits for each sample are shown in SI Figure 4.1–4.6. TRMC measurement error is dominated by the error in measuring film absorption and errors associated with sample inconsistencies. The error shown for the yield data in Figure 2 is estimated by taking an average yield for three replicate films for each sensitizer and then taking the standard deviation of the mean.

### Time-Resolved Photoluminescence

Optical excitation with ca. 100 ps pulses at 650 nm was supplied by an NKT continuous fiber laser (SuperK EXU-6-PP) with 2.69 MHz repetition rate. A 10 nm band-pass filter was used to reduce the spectral bandwidth of the excitation beam. A Hamamatsu 300–900 nm (C10910-04) streak camera was used to collect time-resolved PL spectra. Instrument response was captured by scattering some excitation light into the detector using ground glass in the sample position. Transients were analyzed at the wavelength of the maximum PL intensity for each film.

### Transient Absorption

Transient absorption experiments were conducted using a Coherent Libra Ti:sapphire laser with a rep rate of 1 kHz and an 800 nm fundamental wavelength (150 fs pulse width). The pump wavelengths (600, 650, and 700 nm) were generated in an optical parametric amplifier (TOPAS-C, Light Conversion), while the probe pulse (λ_*probe*_ = 440–800 nm) was generated by focusing a small portion of the 800 nm fundamental into a sapphire crystal. Pump and probe pulses were focused at the sample and spatially overlapped. A mechanical delay stage is used to delay the probe relative to the pump, where the range of the experiment was −2 ps to 5.3 ns. A small portion of the probe was picked off before the sample and directed to a reference detector to reduce noise to *<*0.1 mOD. The changes in the probe spectrum were collected by a fiber optic coupled multichannel spectrometer with a CMOS sensor, while the pump was modulated at 500 Hz by a chopper. Helios and Surface Xplorer software (Ultrafast Systems) were used to collect and analyze the data, respectively. The data were chirp corrected.

### Standard Errors

Standard errors reported in this Letter are from averaged repeated measurements from each experiment. In doing so, we report the averaged value and the standard deviation of the mean from the repeated measurements as the experimental value and the error for those experiments. If a quantity is determined from multiple experimental values, such as Δ*G*_*CT*_, then the error reported is the propagated error from each experimental value, combined in quadrature.

## Supporting Information Available

Absorption and emission characteristics of all samples and control samples, cyclic voltammograms for all sensitizer and host molecules, microwave conductivity transients for all samples and parameters from the global fits to each data set, photoluminescence quenching and photoluminescence quantum yield, FRET discussion and calculations, femtosecond transient-absorption characterization and discussion of every sensitizer molecule in polystyrene and PCBM at varying excitation wavelengths, and time-resolved photoluminescence characterization of every sensitizer molecule; competing hypotheses for low free-charge yield: triplet states and delocalized PCBM excited states (PDF)
