# Supplementary material for: Missing Excitons: How Energy Transfer Competes with Free Charge Generation in Dilute-Donor/Acceptor Systems
Source: ACS Energy Lett. 2024 Feb 8;9(3):896–907. doi: 10.1021/acsenergylett.3c01969 (PMC10928706; doi:10.1021/acsenergylett.3c01969)
Supplement: Supplementary file 1 — nz3c01969_si_001.pdf [file nz3c01969_si_001.pdf]

Supporting Information for: Missing Excitons: How Energy Transfer Competes With Free Charge Generation in Dilute-Donor/Acceptor Systems

Joshua M. Carr[1], Melissa K. Gish[2], Obadiah G. Reid[2,3,*], and Garry Rumbles[1,2,3,4,*]

[1]University of Colorado Boulder, Materials Science and Engineering Program, Boulder CO 80303
[2]National Renewable Energy Laboratory, Chemistry and Nanoscience Center, Golden CO 80401
[3]University of Colorado Boulder, Renewable and Sustainable Energy Institute, Boulder CO 80303
[4]University of Colorado Boulder, Department of Chemistry, Boulder CO 80303
[*]Corresponding author, Obadiah.reid@colorado.edu
[*]Corresponding author, Garry.rumbles@nrel.gov

Contents

1 Absorption and Photoluminescence Spectroscopy **2**
1.1 Evidence of sensitization of thin films 3
1.2 Absorption and emission of sensitizers 4
1.3 Spectral overlap of sensitizer emission and PCBM absorption 5
1.4 Photoluminescence quenching spectra and calculations 7
1.4.1 Discussion of photoluminescence quenching trends 8

2 Electrochemical Characterization **9**

3 TRMC Transients, Global Fits, and Table of Fitting Parameters with Discussion **10**
3.0.1 TRMC Discussion 17

4 Photoluminescence Quantum Yield Experiments and Calculations **19**
4.0.1 Discussion of PLQY experiments and calculations 22

5 Förster Resonance Energy Transfer Calculations and Relevant Plots **23**

6 Time-resolved Photoluminescence Transients with Fits and Parameters **27**

7 Transient Absorption Spectra and Excitation Dependent Experiments **29**

8 Do Triplet States Quench Free Charge Yield? **32**

9 Do Delocalized Excitons Explain Yield Enhancements Observed for Direct PCBM Excitation? **33**

1 Absorption and Photoluminescence Spectroscopy

The following absorption and photoluminescence spectra are provided for full characterization of each sensitizer in an inert polystyrene host compared to the accepting PCBM host. In addition, much of the experimental work exhibited in the main text is only for one of a series of sensitizers used in the work. This section is provided for further information on the nature of each sensitizer and how the conclusions about general trends discussed in the main text were drawn.

The discussion of the nature of the sensitization of these thin films is given in more detail in the red sensitizer work from Carr et al. Briefly though, we argue that the minimal red shifting and broadening of the sensitizer absorption from the inert polystyrene host film to the accepting PCBM host film results in a successfully sensitized film for our experimental purposes. The typical optical evidence observed for phthalocyanines and squaraines due to aggregation are absent in the following spectra. For more information see Carr et al. for the full description.[1]

1.1 Evidence of sensitization of thin films

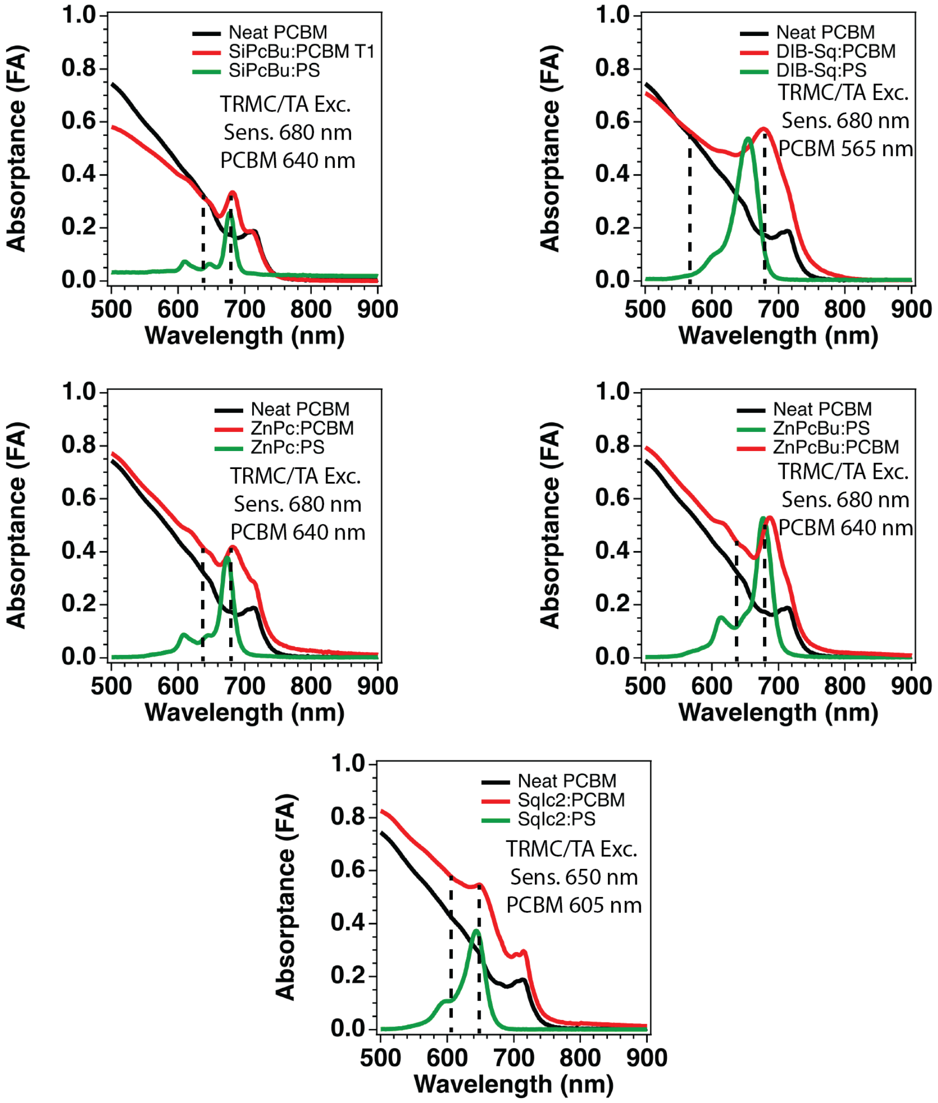

Figure S 1.1: Fractional absorptance spectra of all blue sensitizers at 0.005 mol kg^{-1} in polystyrene (green trace) and in PCBM (red trace) compared to the neat PCBM fractional absorptance (black trace). In this work, we call these sensitizers "blue" because of the energy at which their peak absorption is located, which is to the blue side of the PCBM absorption onset. Excitation wavelengths for the primary TRMC/TA experimental results in the main text are given inset on each spectrum and denoted by the black dashed lines; these are the primarily sensitizer excitation experiments and primarily PCBM host excitation experiments.

1.2 Absorption and emission of sensitizers

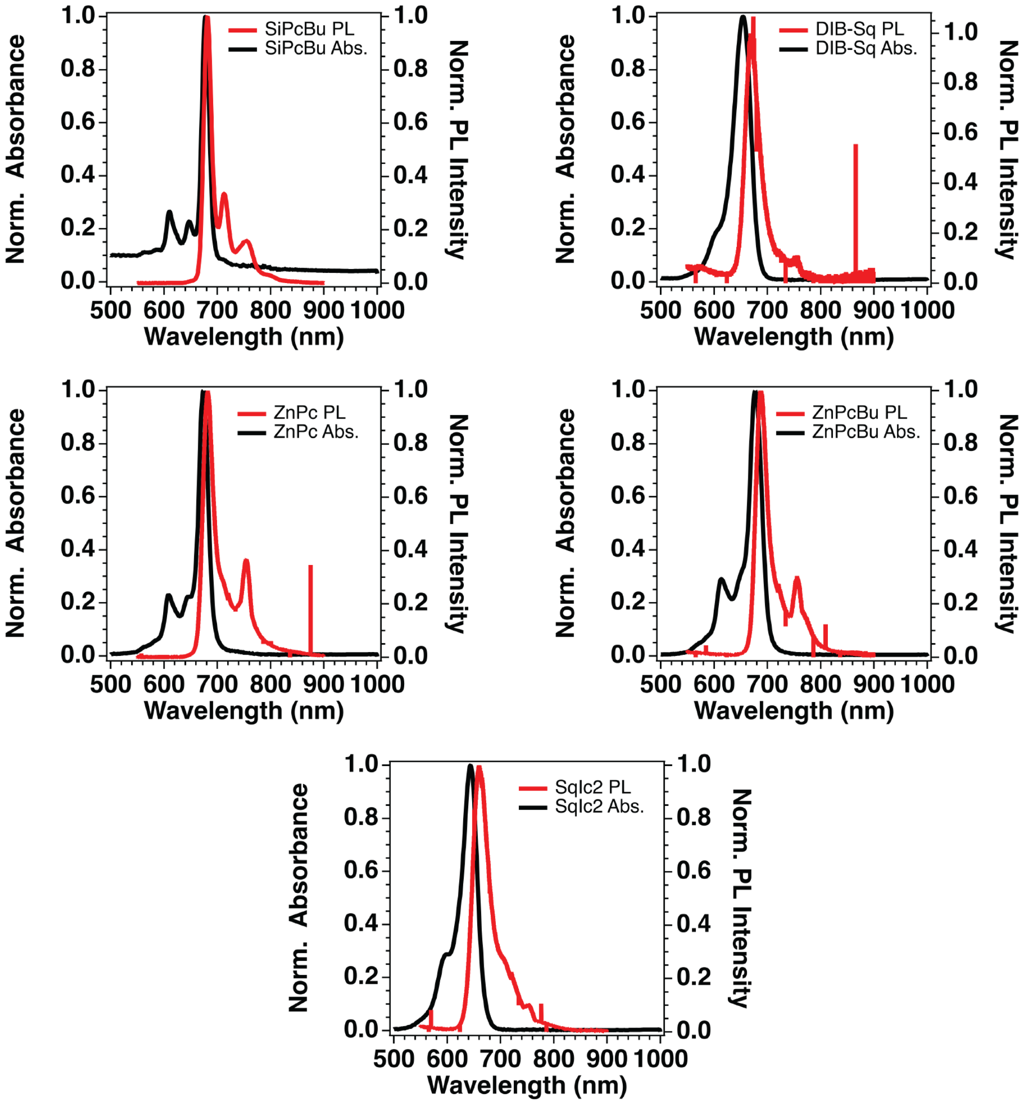

Figure S 1.2: Normalized absorption (black trace) and normalized emission (red trace) for each sensitizer at 0.005 mol kg^{-1} in a polystyrene inert host. Samples are all excited far away from their emission at wavelengths between 350-400 nm. Of particular interest is the consistent trend of a very small Stokes shift ($<$ 10 nm) for each sensitizer which is what allows for significant spectral overlap of these donors with the absorption of the PCBM host.

1.3 Spectral overlap of sensitizer emission and PCBM absorption

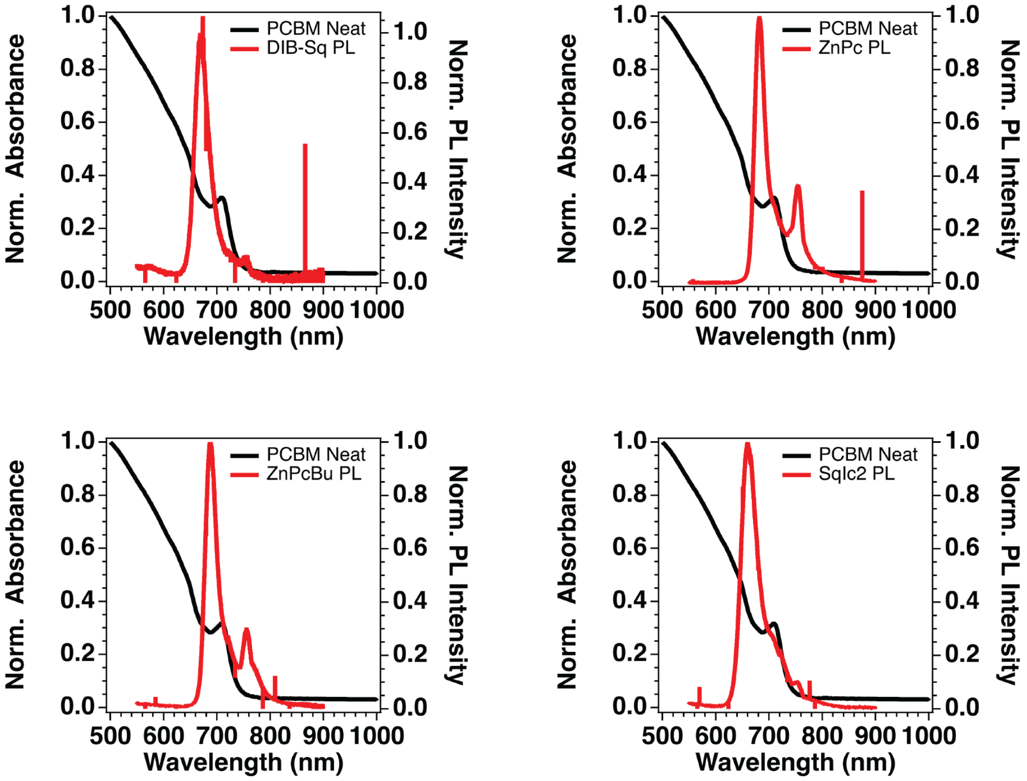

Figure S 1.3: Normalized absorption of neat PCBM film at 500 nm compared to normalized emission of each sensitizer at 0.005 mol kg^{-1} in a polystyrene inert host. SiPcBu is omitted from here as the same plot reproduced for that samples is located in the main text in **Figure 1**. This figure serves to demonstrate the moderate spectral overlap of PCBM absorption and sensitizer emission in the region between 650 - 750 nm, which is responsible for the ultrafast energy transfer processes now available for the blue sensitizers which were not present in the previous red sensitizer work in Carr et al.[1]

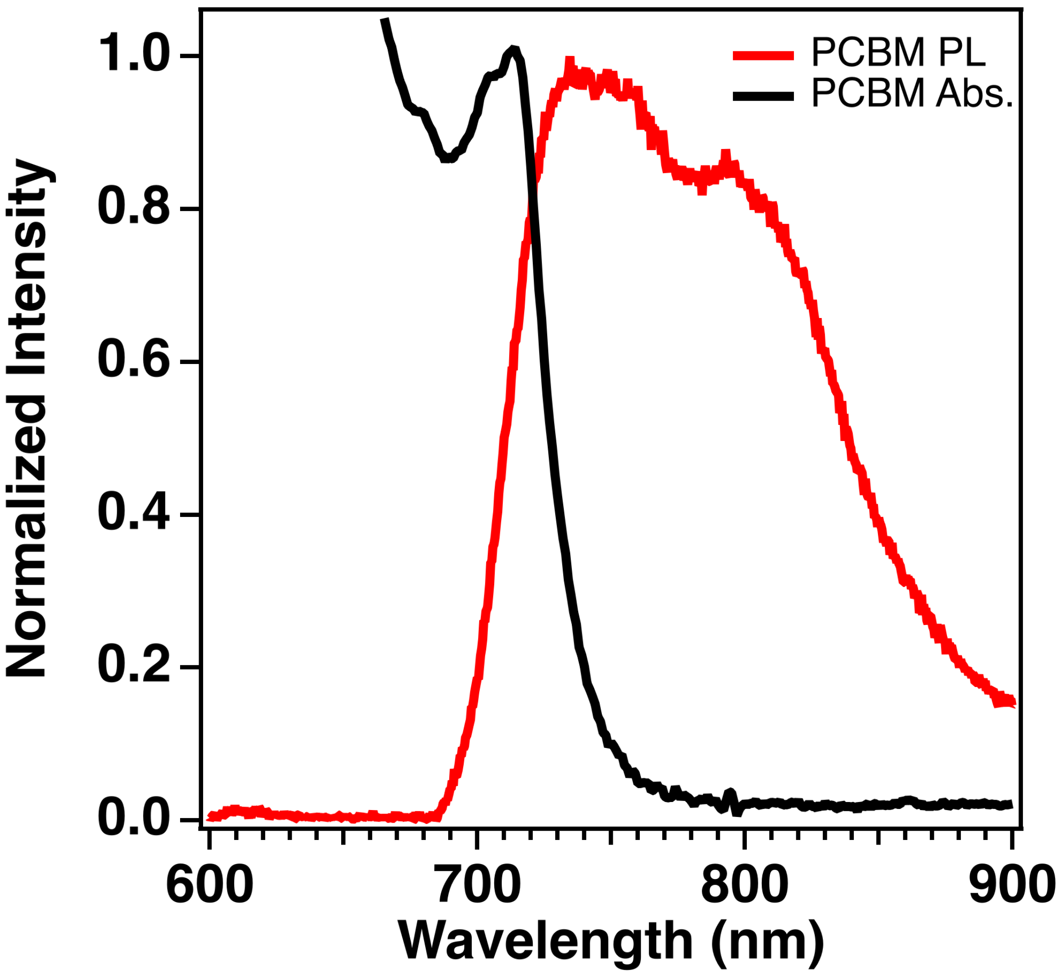

Figure S 1.4: Normalized absorption (black trace) at 715 nm and normalized emission (red trace) for neat PCBM. Emission is collected from excitation at 500 nm. The overlap of the absorption and emission at ca. 730 nm is used as a conservative estimate of the lowest lying exciton energy in the molecular system for the purposes of the driving force calculations.

1.4 Photoluminescence quenching spectra and calculations

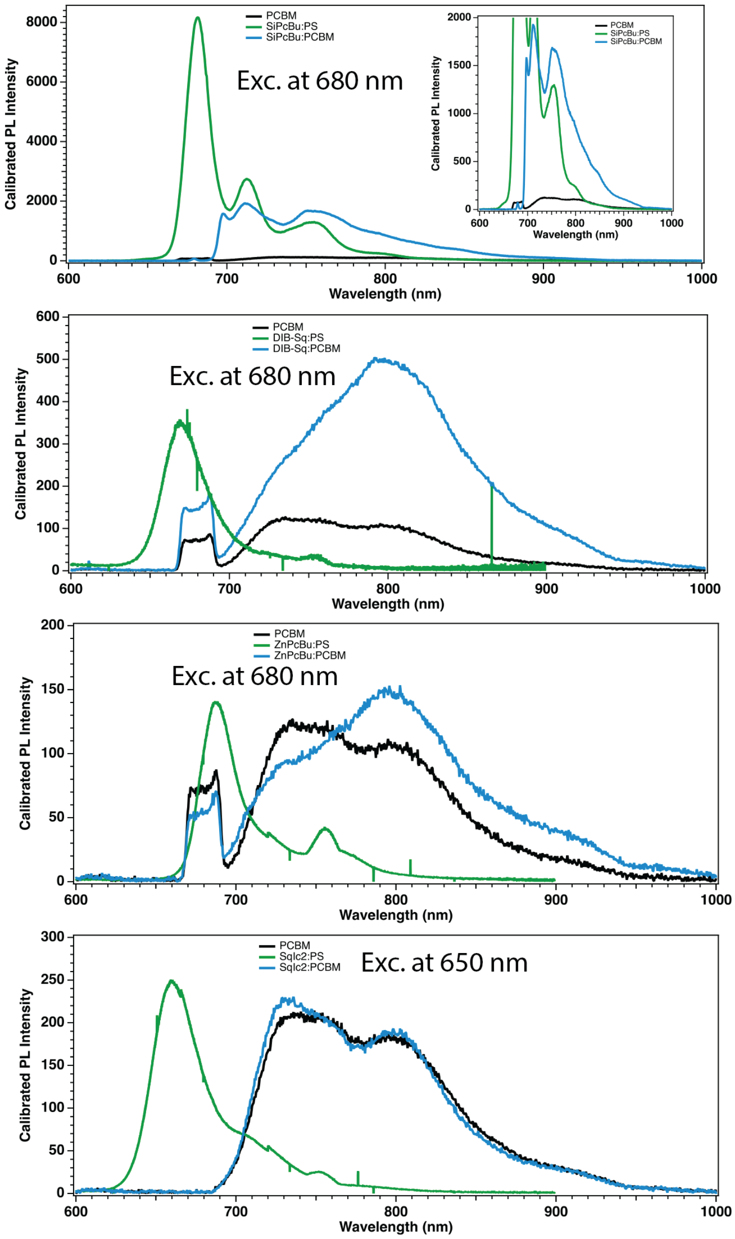

Figure S 1.5: Detector intensity calibrated photoluminescence spectra for all other sensitizers except ZnPc (located in main text **Figure 4**). The green traces are the sensitizer emission from the polystyrene inert host excited far away from the emission feature between 350-400 nm. The black traces are neat PCBM emission and the blue traces are the emission from exciting the sensitized PCBM accepting host films, both at the same wavelengths corresponding to the TRMC/TA excitations on the peak of the sensitizer absorption. The peaks in the blue and black traces at those excitation wavelengths are some excitation light making it to the detector even with the 700 nm long-pass filter in place.

1.4.1 Discussion of photoluminescence quenching trends

We conducted photoluminescence quenching (PLQ) experiments on each sensitizer to verify that we indeed had quenched the excited states of the sensitizer and are left with mostly emission character from the PCBM host. For all samples but SiPcBu, we find that the same trends discussed in the main text are present here, that is, efficient quenching of the sensitizer:PS emission features in sensitizer:PCBM emission, and in addition exhibits a qualitatively different emission spectrum compared to the neat PCBM, whereby a broad emission features grow in, that we contribute to CT emission. This is most obvious for both DIB-Sq and ZnPcBu, both of which show new qualitative CT emission features centered at 800 nm, of which are not present in the neat PCBM emission spectrum. The SqIc2 sensitizer shows no presence of the sensitizer emission features present, however, there is not much change from the PCBM emission.

SiPcBu is different from the other sensitizers for a couple reasons: (1) SiPcBu is the lowest driving force sample (-200 meV of driving force) and as such we would expect to have the lowest PLQ ratio out of all samples. (2) SiPcBu has a substantial PLQY (ca. 33%, see below in **SI Section 4**) so for similar excitation densities has much more intensity compared to the other sensitizers and neat PCBM emission. For example, SiPcBu has nearly 8000 calibrated counts, meanwhile none of the other sensitizers or PCBM approach 1000 calibrated counts. With a PLQ ratio of 90%, you might expect SiPcBu peak emission to be at minimum 800 counts in the same environment, independent of any other increases to it's emission. Qualitatively observing the reduction of SiPcBu emission is difficult since most of the emission from the SiPcBu:PCBM film is coming the SiPcBu emission and in addition, there are EET and PET processes occurring simultaneously.

Given the above discussion, we approximate the PLQ ratio from this system with the equation:

$$PLQ = 1 - \left(\frac{PL_{PCBM}/A_{PCBM}}{PL_{PS}/A_{PS}} \right) \tag{1.1}$$

where we take the ratio of the calibrated counts for each spectrum normalized by %A at the excitation wavelength. The resulting PLQ ratios are given in the table below for each sensitizer, including ZnPc from the main text. We approximate the PL quenching at only one wavelength instead of integrating over the entire spectrum, as would typically be done, since there are additional effects from energy transfer and CT emission.

Table S 1.1: PLQ ratios for each sensitizer calculated using the above equation and spectra. The PLQ ratios are determined at 730 nm for all sensitizers *except* SiPcBu, since we know that it is largely accounting for the PL in the SiPcBu:PCBM film, we elect to determine this instead at the peak of the SiPcBu emission overlapping with PCBM at 710 nm.

Sensitizer	PLQ Ratio
SiPcBu at 710 nm	0.52
DIB-Sq at 730 nm	0.97
ZnPc at 730 nm	1
ZnPcBu at 730 nm	0.94
SqIc2 at 730 nm	0.88

2 Electrochemical Characterization

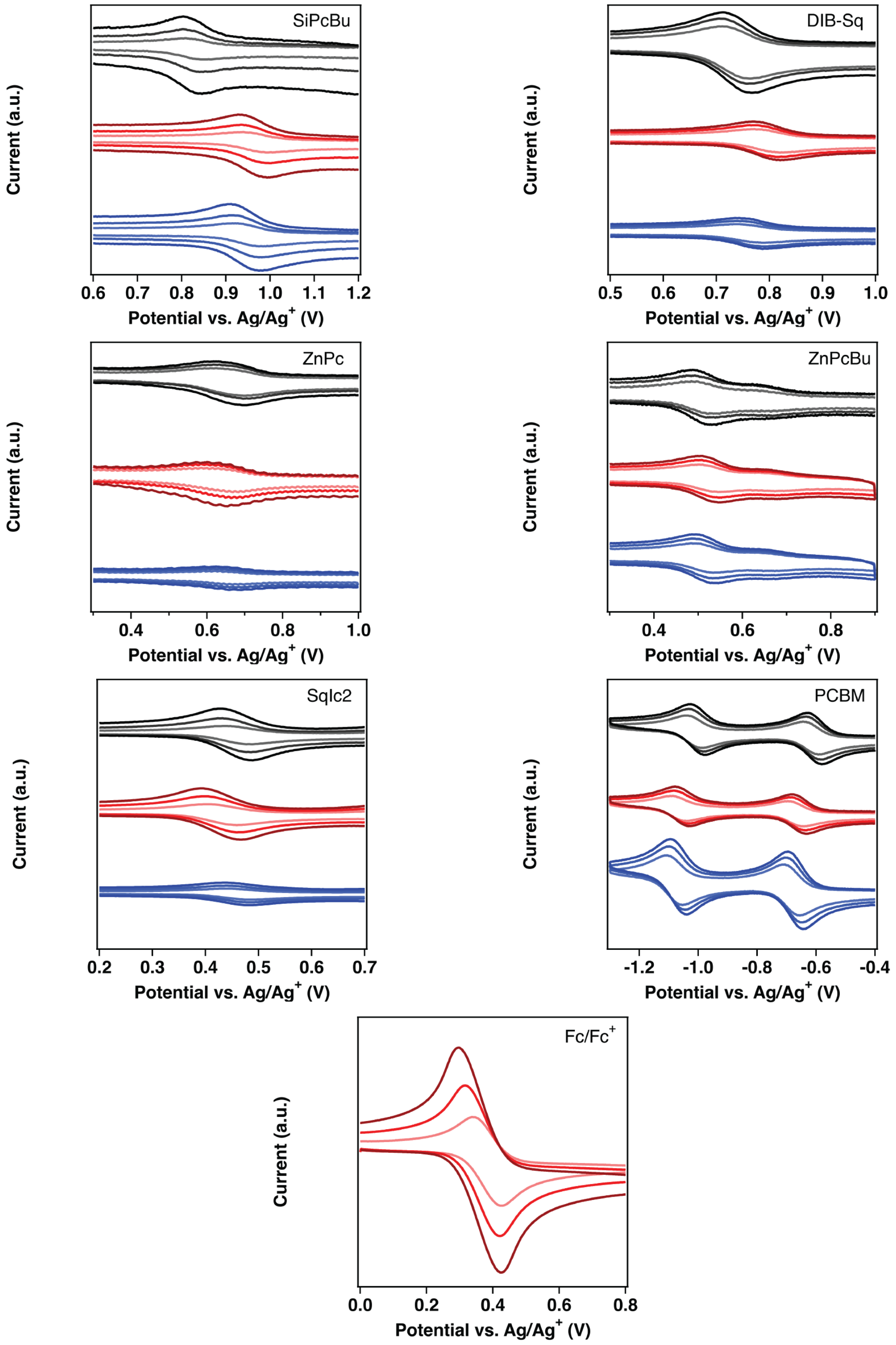

Figure S 2.1: Triplicate cyclic voltammetry scans for each sensitizer, PCBM, and the ferrocene (Fc) standard, measured at varying scan rates of 100, 150, and 200 mV/s as denoted by the increasing shades of color (lighter is slowest scan rate, darkest is fastest). Each set of colors is for a different independent solution at the same 2 mM analyte concentration. All $E_{1/2}$ values are given in the main text in **Table 1** with error estimates.

3 TRMC Transients, Global Fits, and Table of Fitting Parameters with Discussion

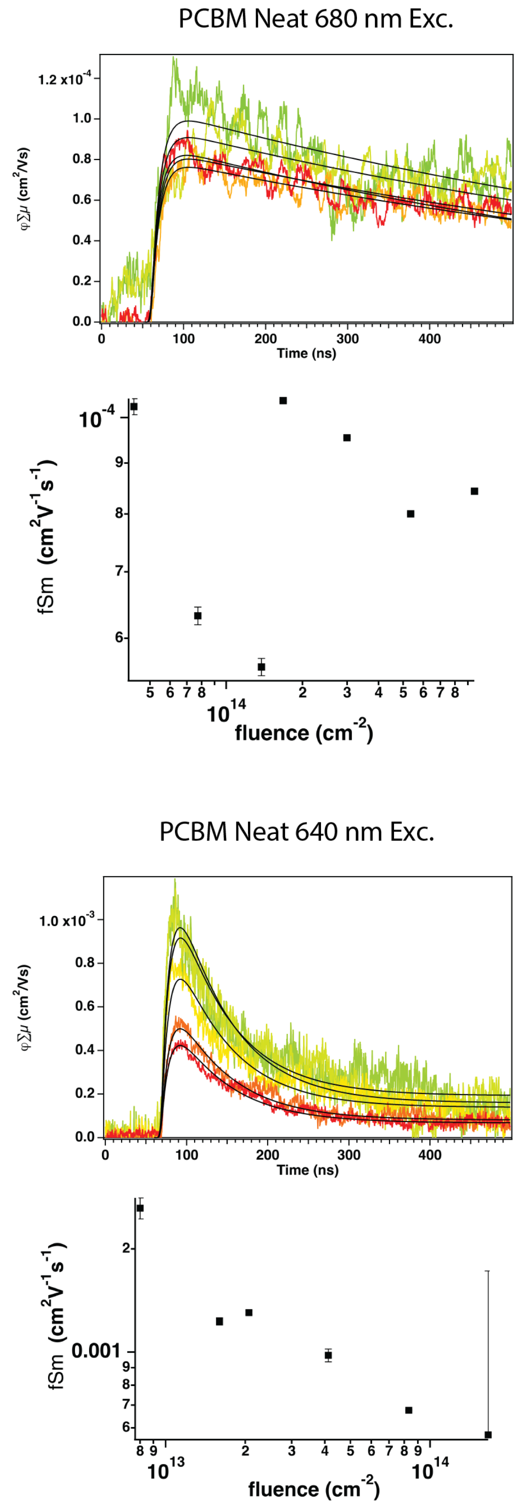

Figure S 3.1: TRMC transients for neat PCBM spray coated film at two wavelengths, 680 nm and 640 nm, including fits (black traces). All transients in the figure at varying fluences ranging from 10^{13}-10^{15} photons/cm^2 as shown in the fluence plot.

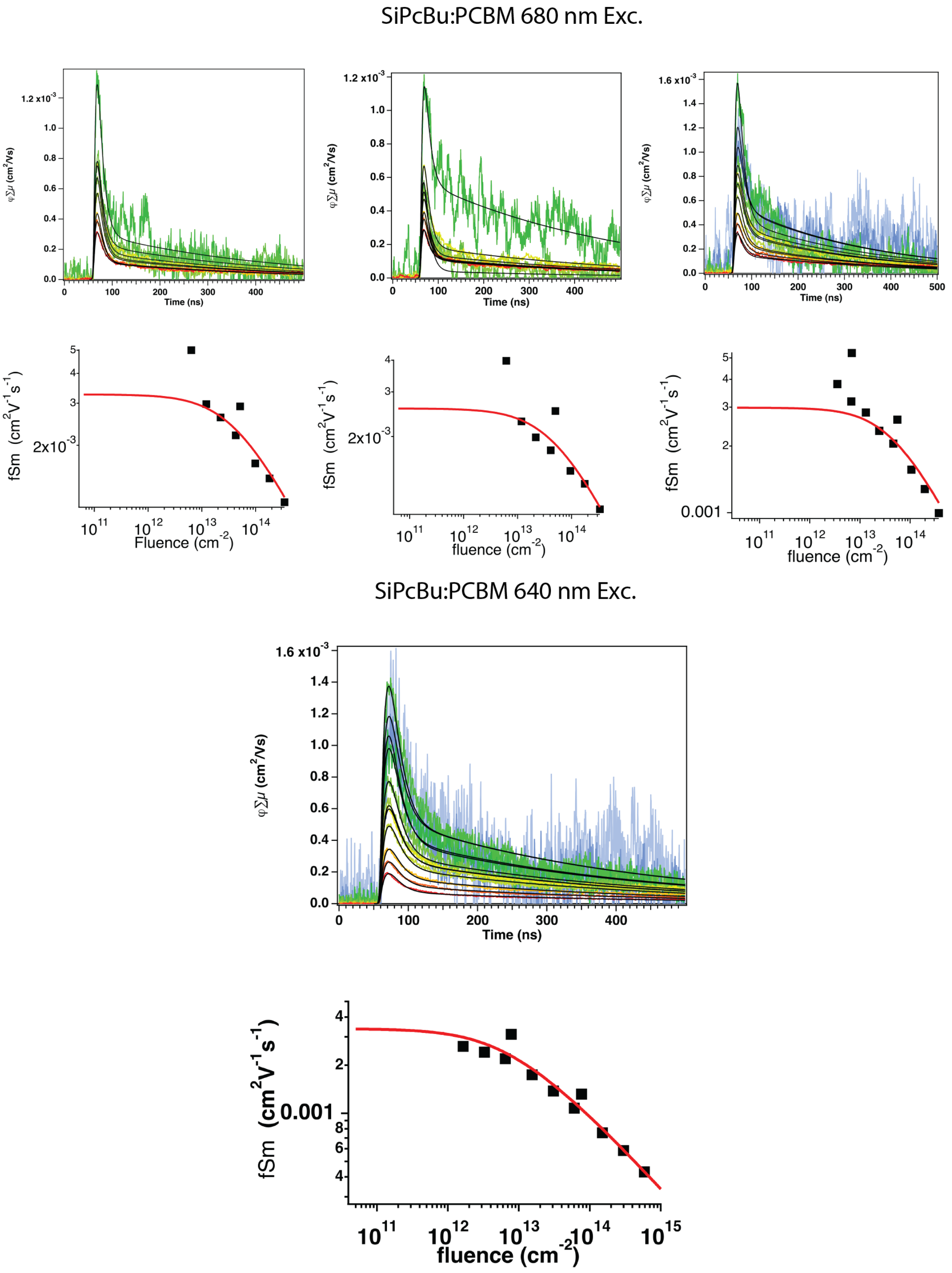

Figure S 3.2: TRMC transients for SiPcBu:PCBM spray coated films at two wavelengths, 680 nm and 640 nm, including fits (black traces). All transients in the figure at varying fluences ranging from 10^{13}-10^{15} photons/cm^2 as shown in the fluence plot.

DIB-Sq:PCBM 680 nm Exc.

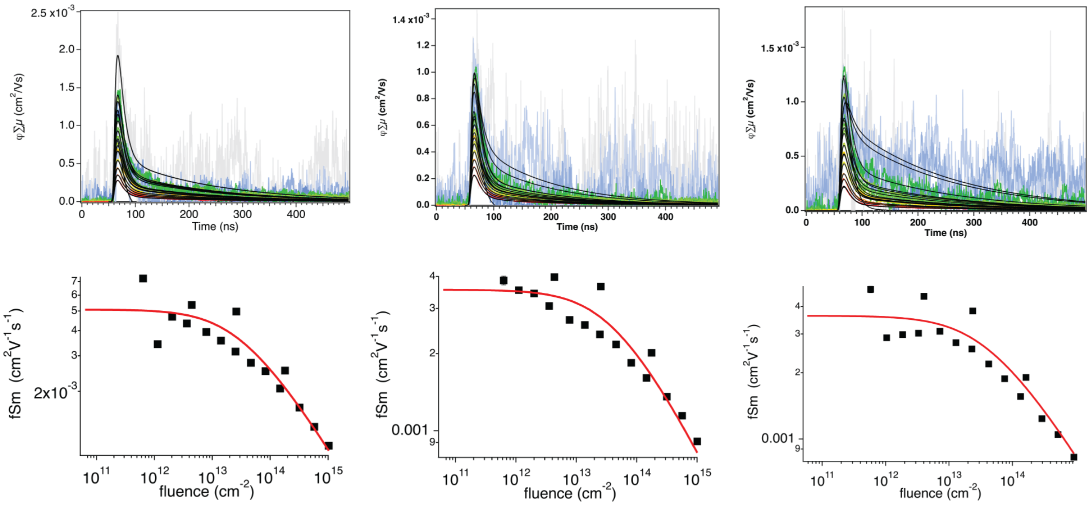

DIB-Sq:PCBM 565 nm Exc.

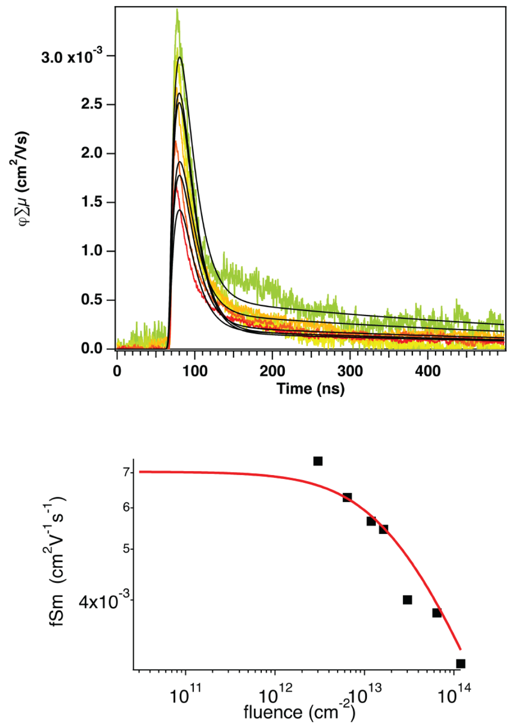

Figure S 3.3: TRMC transients for DIB-Sq:PCBM spray coated films at two wavelengths, 680 nm and 565 nm, including fits (black traces). All transients in the figure at varying fluences ranging from 10^{12}-10^{15} photons/cm^2 as shown in the fluence plot.

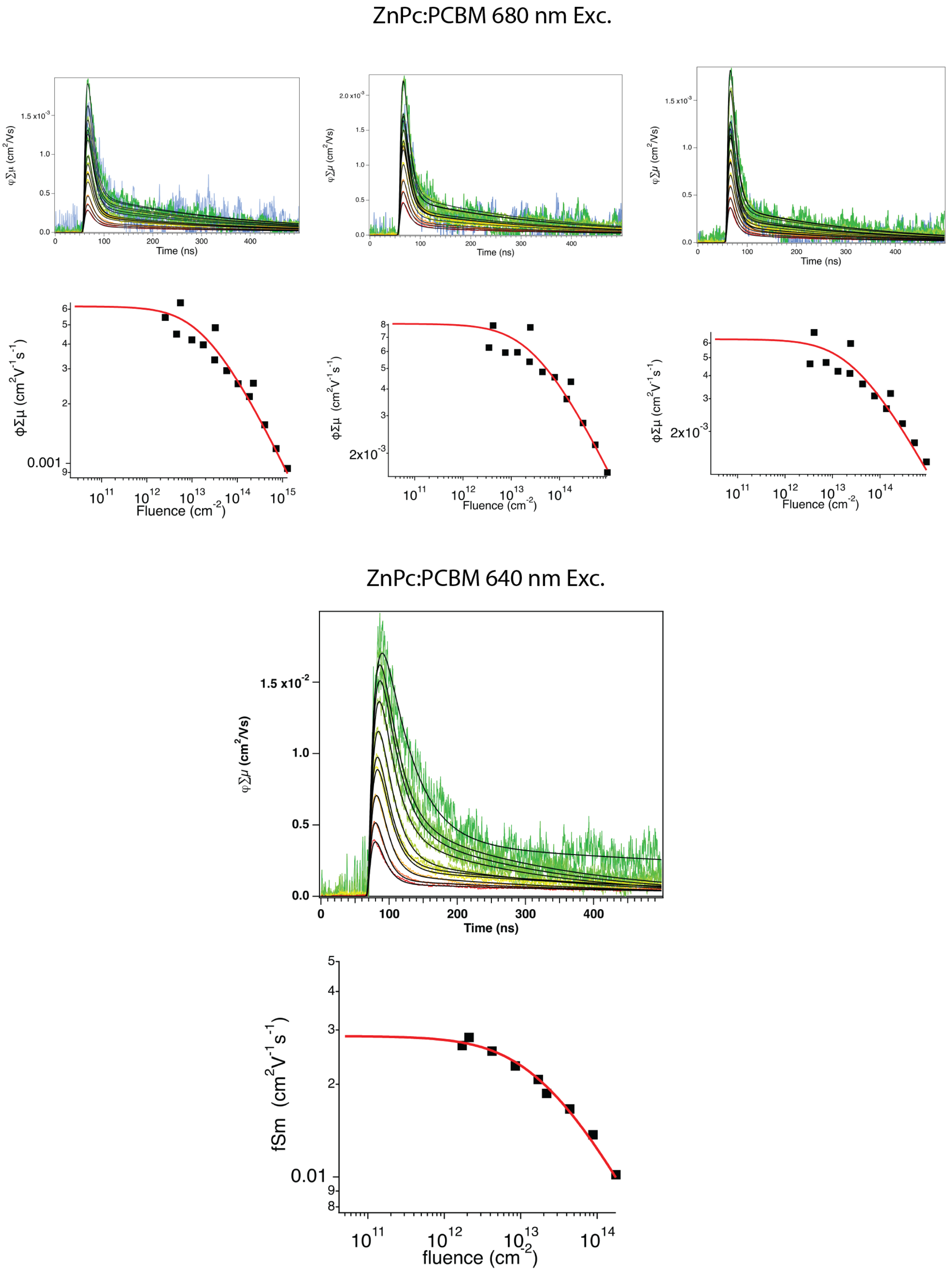

Figure S 3.4: TRMC transients for ZnPc:PCBM spray coated films at two wavelengths, 680 nm and 640 nm, including fits (black traces). All transients in the figure at varying fluences ranging from 10^{12}-10^{15} photons/cm^2 as shown in the fluence plot.

ZnPcBu:PCBM 680 nm Exc.

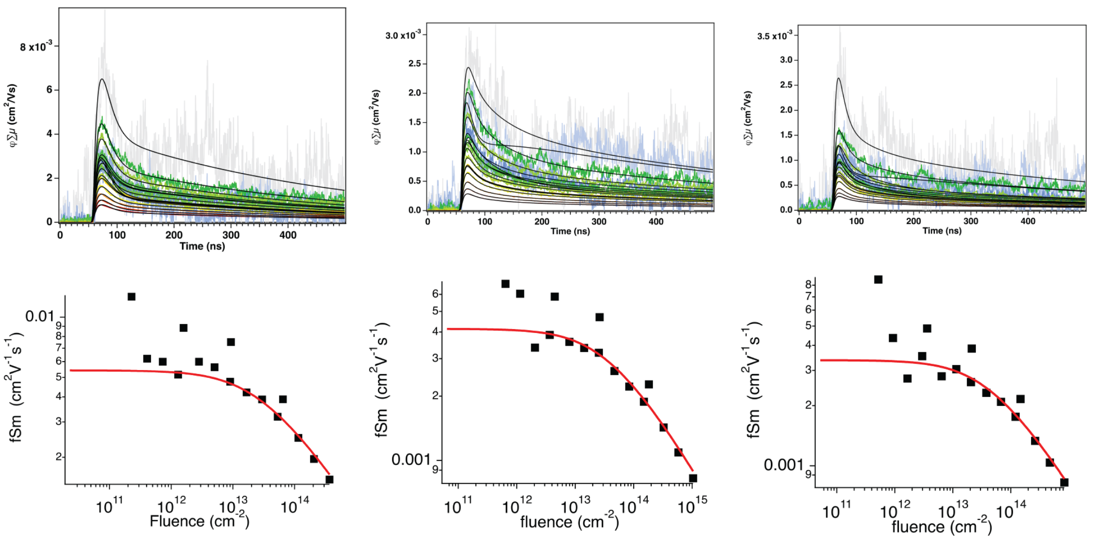

ZnPcBu:PCBM 640 nm Exc.

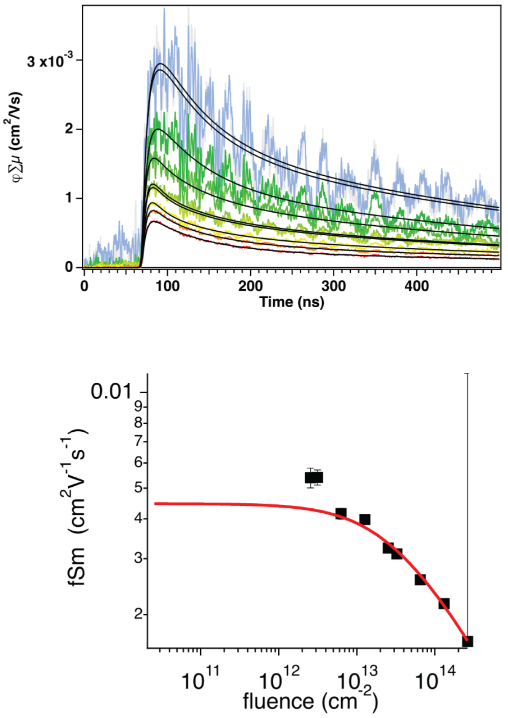

Figure S 3.5: TRMC transients for ZnPcBu:PCBM spray coated films at two wavelengths, 680 nm and 640 nm, including fits (black traces). All transients in the figure at varying fluences ranging from 10^{12}-10^{15} photons/cm^2 as shown in the fluence plot.

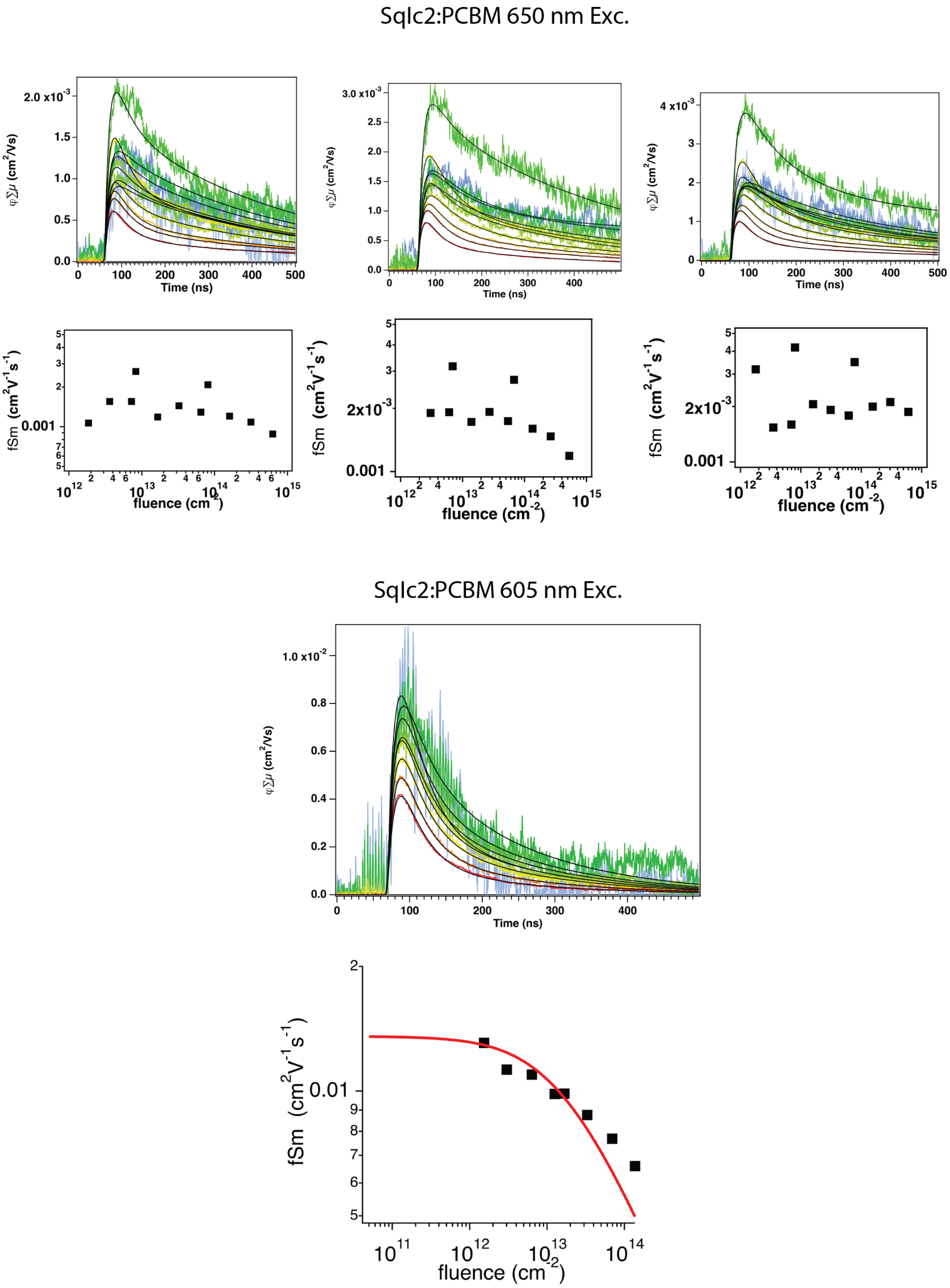

Figure S 3.6: TRMC transients for SqIc2:PCBM spray coated films at two wavelengths, 650 nm and 605 nm, including fits (black traces). All transients in the figure at varying fluences ranging from 10^{12}-10^{15} photons/cm^2 as shown in the fluence plot.

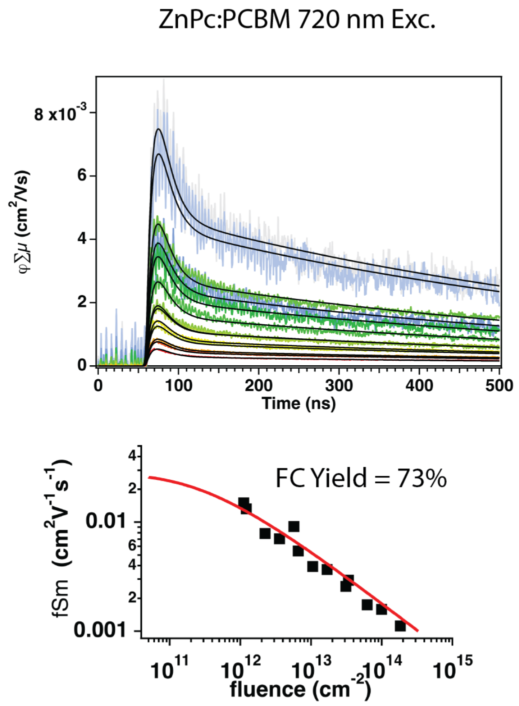

Figure S 3.7: TRMC transients and yield-mobility product as a function of fluence for ZnPc:PCBM spray coated film at 720 nm, including fits (black traces). All transients in the figure at varying fluences ranging from 10^{12}-10^{15} photons/cm^2 as shown in the fluence plot. The FC yield quoted inset on the figure is determined from the exciton-charge annihilation model as described in the discussion below. This experiment is conducted as a test of whether delocalized excitons in the PCBM at higher excitation energies between 565-640 nm are the cause of increased FC yield. 720 nm excitations are much closer to the localized PCBM exciton and show similar yield that is quoted for the excitation on the same sample at 640 nm as is discussed in the main text.

Table S 3.1: Table of all TRMC global fit parameters for each film for each sensitizer. τ is the time constant while A is the amplitude for each exponential, indexed 0 and 1. For each sensitizer, there are T1-T3 samples which are triplicate films for the TRMC measurements at the sensitizer peak absorption (650-680 nm) while the final row for each species is a final independent film for the primarily PCBM excitation transient parameters (565-640 nm), except for ZnPc, which has two independent excitation dependent experiments at both 640 and 720 nm.

Species	Sample	$\tau_0(s^{-1})$	$\tau_1(s^{-1})$	A_0	A_1
SiPcBu	T1	5.02E-09	4.45E-07	0.001	0.0001
	T2	4.16E-09	4.08E-07	0.001	0.0001
	T3	4.65E-09	3.88E-07	0.001	0.0001
	640 nm	1.01E-08	3.12E-07	0.0004	0.00008
DIB-Sq	T1	5.03E-09	1.54E-07	0.001	0.0001
	T2	4.71E-09	1.19E-07	0.001	0.00005
	T3	5.97E-09	1.76E-07	0.001	0.00005
	565 nm	5.28e-09	1.67e-07	0.006	0.0005
ZnPc	T1	5.80E-09	3.03E-07	0.001	0.0001
	T2	5.34E-09	2.95E-07	0.002	0.0001
	T3	5.00E-09	2.74E-07	0.001	0.0001
	640 nm	4.19E-08	1.07E-06	0.02	0.004
	720 nm	1.06E-08	6.91E-07	0.0009	0.0003
ZnPcBu	T1	1.39E-08	4.25E-07	0.001	0.0005
	T2	4.75E-09	5.59E-08	0.0005	0.0001
	T3	4.72E-09	5.85E-08	0.001	0.0001
	640 nm	2.54E-09	5.75E-08	0.002	0.0002
SqIc2	T1	5.33E-08	1.39E-06	0.001	0.002
	T2	6.37E-08	1.56E-06	0.0003	0.002
	T3	5.95E-08	1.26E-06	0.001	0.002
	605 nm	2.72E-08	1.38E-07	0.005	0.002
PCBM	680 nm	3.44E-08	3.83E-07	0.0002	0.0001
	640 nm	7.65E-08	4.14E-05	0.0005	0.00004

3.0.1 TRMC Discussion

Film photoconductivity for this work is determined by the following: (1) TRMC transients are collected as a function of light intensity for each sample in the series to ensure that the response is linearly correlated. (2) All transients are globally fit with biexponential functions convoluted with the 7 ns cavity response. (3) The resulting yield-mobility product ($\phi\Sigma\mu$) peak value is normalized by the fraction of absorbed photons in the film. The resulting fluence-dependent ($\phi\Sigma\mu$) curves are then fit with an exciton-charge annihilation (ECA) model, which has previously been used to fit charge yields as a function of laser fluence where parasitic processes occur at large laser fluence causing a reduction in apparent charge yield. This has been described in detail by Ferguson et al., Reid et al., Hodgkiss et al., and O'Connor et al.[2–5]. The exact model used in this work is from O'Connor et al.

Mechanistically, we use the ECA model to fit the $\phi\Sigma\mu$ vs. fluence plots to extract absolute yield values at low fluences (10^{10}-10^{11} photons/cm^2) where the parasitic annihilation processes no longer occur. The process by which we arrive at the

yield values in this work, and as described in the main text are as follows. We fit the ZnPc:PCBM film excited at both 680nm (primarily sensitizer absorption) and 640nm (primarily PCBM absorption) with an assumed PCBM electron mobility of 0.04 $cm^2V^{-1}s^{-1}$, known from prior work[6, 7] which allows us to extract a yield and ECA rate constant, γ_{ECA} of 4.89 x 10^{-8} cm^3 s^{-1} and 4.18 x 10^{-8} cm^3 s^{-1} respectively. Since the ZnPc:PCBM film produces the largest yield of charges, we then constrain the remaining samples to those γ_{ECA} values for the respective excitation wavelengths, either 4.89 x 10^8 s^{-1} for the primarily sensitizer excitations or 4.18 x 10^8 s^{-1} for the primarily PCBM excitations, and fit the data for just the free charge yield. For the control experiment of exciting the ZnPc:PCBM film at 720 nm we find an ECA rate constant of 3.32 x 10^{-6} cm^3 s^{-1}, which is consistent with the lower excitation density at the 720 nm excitation wavelength for that film. The choice to constrain the data based on γ_{ECA} from ZnPc:PCBM is because the remaining films produce far less charges than the ZnPc:PCBM and in most cases do not produce charges at a low enough fluence to observe a plateau region in the yield values (see SiPcBu:PCBM for an example).

The main feature of all microwave conductivity transients in this study is the biexponential shape, specifically a fast decay component (ca. 10 $\pm$ 5 ns) most likely associated with a trapping process and a second slower decay component (ca. 500 $\pm$ 100 ns) most likely due to recombination. However, those loss mechanisms occur on a timescale orders of magnitude longer than the charge generation step. In addition, there seems to be no ΔG_{CT} dependence on the lifetime or shape of the transients across all samples in the study, which is consistent with having only mobile electrons in the PCBM host that absorb microwave power for each sensitizer:PCBM combination. We do note that the SqIc2 sample at both excitation wavelengths has a longer lived charge population that is consistent with the neat PCBM charge lifetimes indicating a possibly different kinetic process at the largest driving force, which was also observed for the red sensitizer data in Carr et al.[1] Having observed this a second time at the largest driving force in a series of sensitizers in the PCBM host might indicate that the charge generation process at those largest driving forces, where CT state generation is fastest, is delayed due to the slow FC state rate.

Of final note is that the excitations into primarily PCBM do not show drastically different transient shapes or lifetimes. All of them can be fit with global biexponentials in the same way that the excitations into primarily the sensitizer can be fit. In addition, we notice that for ZnPc:PCBM specifically, which is the sample that benefits the most in terms of recovery of FC yield with the primarily PCBM excitations, that we find an increase in overall charge lifetime (τ_0 increases from ca. 5 ns to 50 ns, τ_1 increases from ca. 300 ns to 1 μs) as well, which could be due to waiting for some exciton diffusion in the PCBM host before charge separation. However, since the transient shape and lifetimes don't qualitatively change and don't drastically change quantitatively over the entire series we conclude that this is consistent with our hypothesis that FC yield in the blue sensitizer system is almost entirely dominated by the PCBM excitations at either wavelength.

4 Photoluminescence Quantum Yield Experiments and Calculations

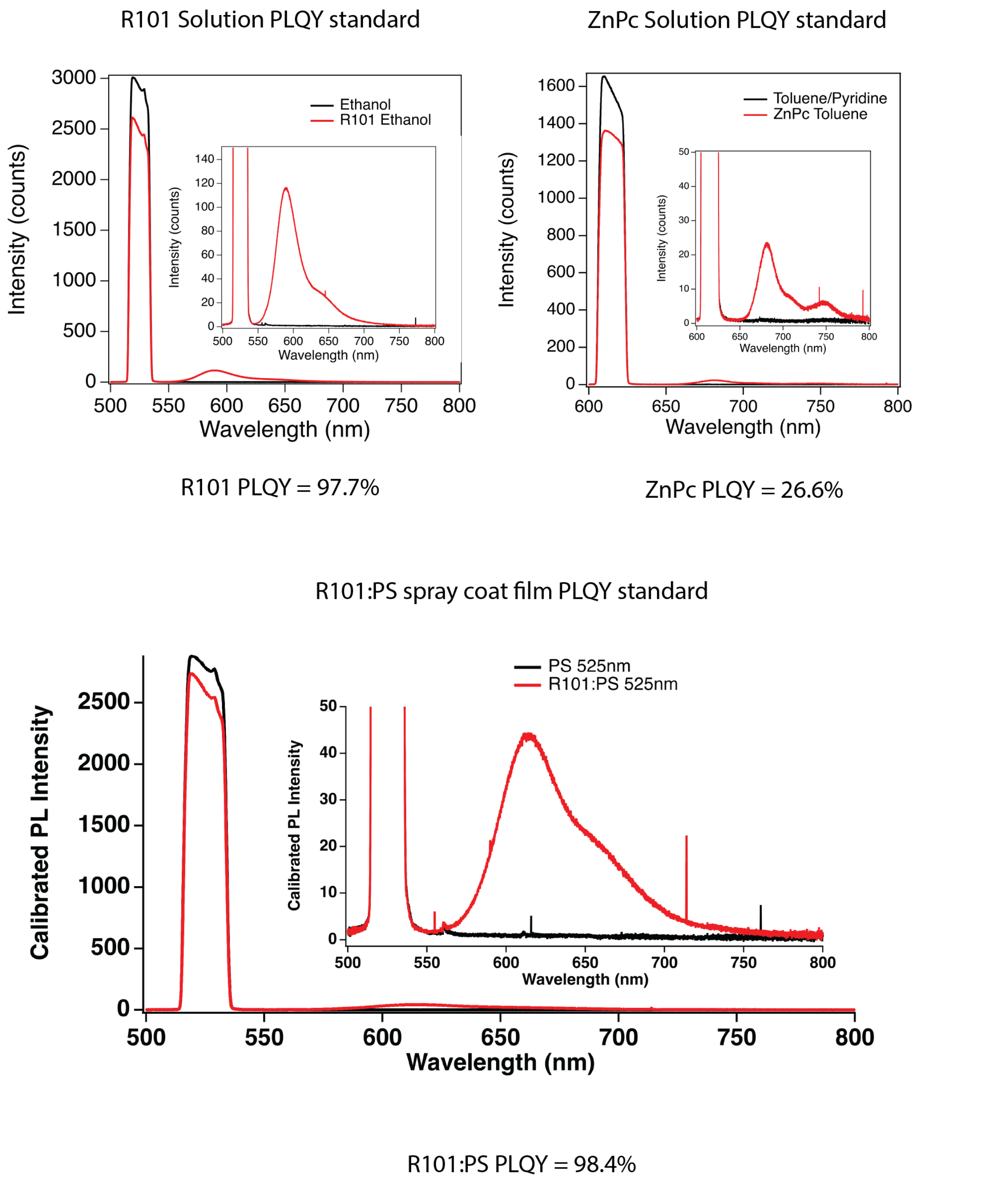

Figure S 4.1: Detector intensity calibrated photoluminescence spectra for calculation of photoluminescence quantum yield (PLQY) which is given here. Top images are of two PLQY standards in solution, rhodamine-101 (R101) dissolved in ethanol (top left) and ZnPc in 99% toluene/ 1% pyridine (top right). Bottom image is of R101 in the same inert polystyrene host that the sensitizers are, as a control PLQY experiment for the films.

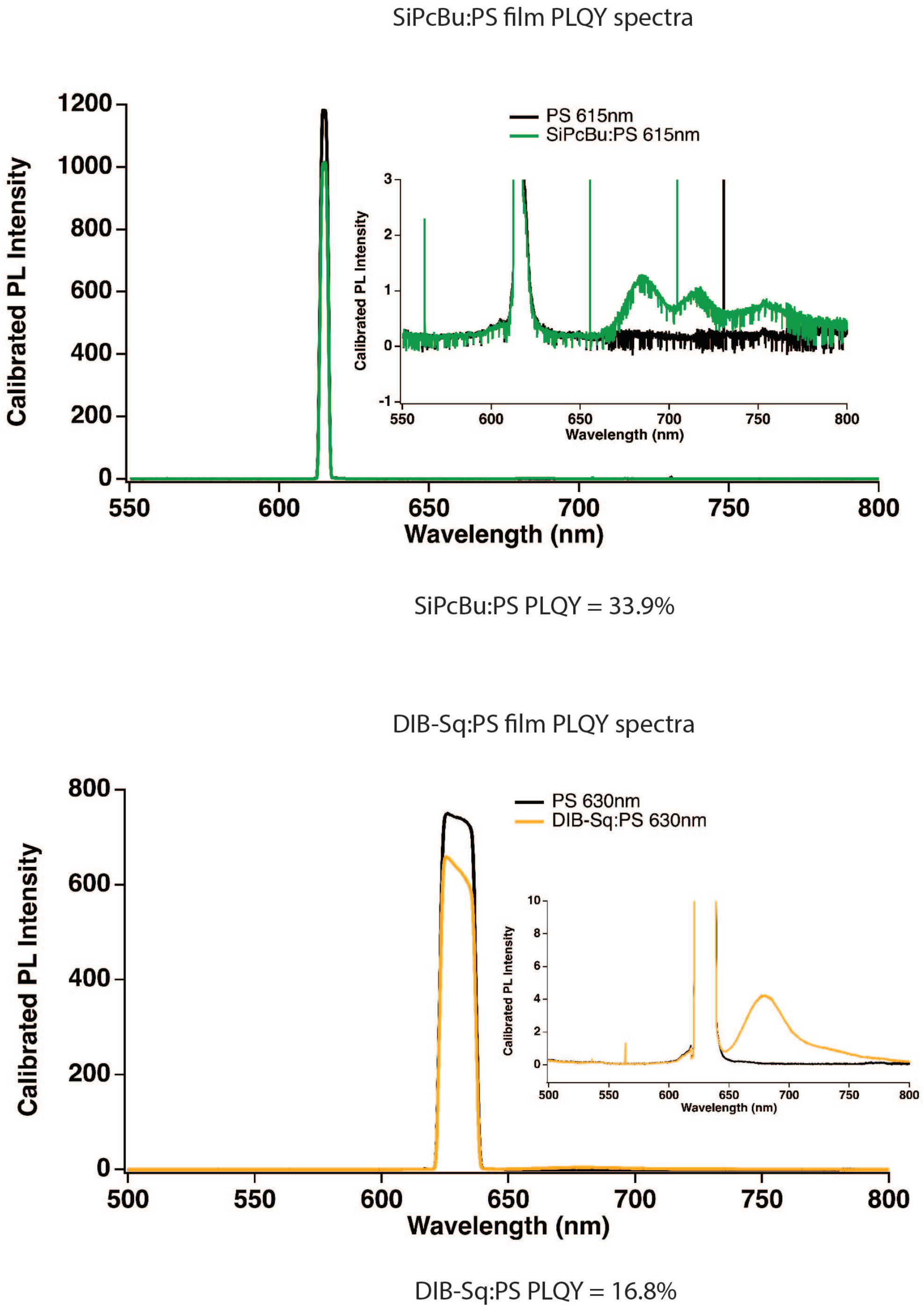

Figure S 4.2: Detector intensity calibrated photoluminescence spectra for calculation of photoluminescence quantum yield (PLQY) which is given here. Top image is for SiPcBu:PS excited at 615 nm. Bottom image is of DIB-Sq:PS excited at 630 nm.

ZnPc:PS and ZnPcBu:PS film PLQY spectra

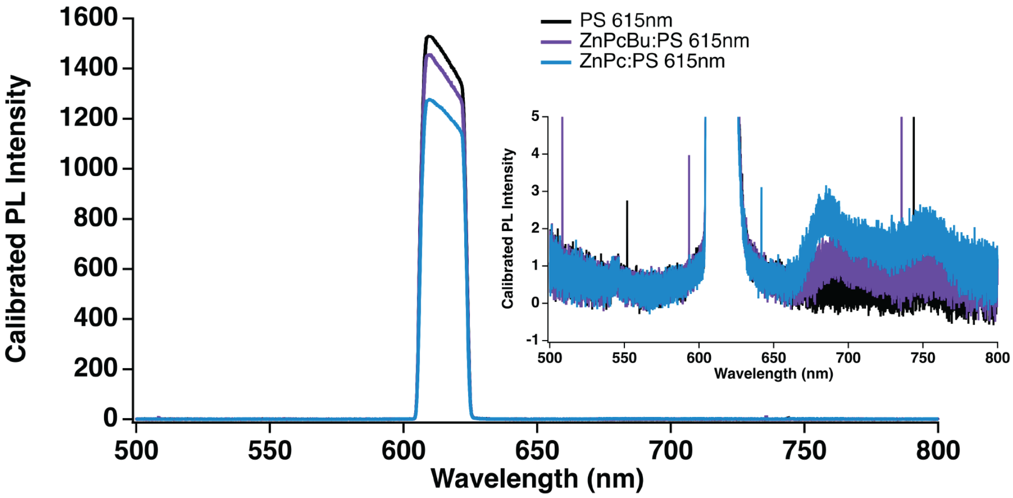

ZnPc:PS PLQY = 5.9%   ZnPcBu:PS PLQY = 9.6%

SqIc2:PS film PLQY spectra

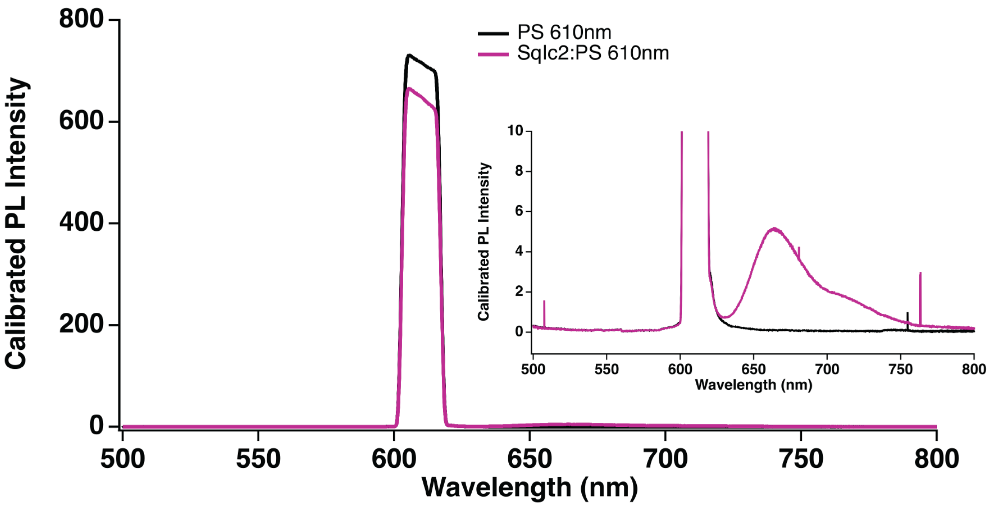

SqIc2:PS PLQY = 33.0%

Figure S 4.3: Detector intensity calibrated photoluminescence spectra for calculation of photoluminescence quantum yield (PLQY) which is given here. Top image is for ZnPc:PS and ZnPcBu:PS excited at 615 nm. Bottom image is of SqIc2:PS excited at 610 nm.

4.0.1 Discussion of PLQY experiments and calculations

Photoluminescence experiments were conducted on each sensitizer in the inert polystyrene host environment to calculate PLQY's for each sensitizer as an isolated molecule in a spray coated film environment. We calculate PLQY in this work towards the ultimate goal of calculating the FRET rate constant for each sensitizer with the PCBM host film in an effort to estimate how much of a contribution FRET has on the entire kinetic scheme. We find in general that PLQY's for the sensitizers can be quite large (ca. 33% for SiPcBu and SqIc2) and vary across the series, with no trends to note. Furthermore, we want to note that these are *absolute* PLQY measurements, not relative measurements. The standard samples are used as controls to estimate the dynamic range of our photoluminescence spectrometer experimental setup and verify the accuracy of it, but the calculations, shown below, are absolute PLQY. The standard PLQY measurements were compared to other previous literature values using the IUPAC technical report to verify the response of the instrument and as a guide for assistance with the experimental setup.[8]

To calculate an absolute and quantitative PLQY experiment, we elected to use an integrating sphere as this provides little loss of incident photons. Second, we calibrate the detector intensity response using the same exposure parameters to be used for the PLQY measurements, and we do so with a known calibration lamp spectrum. Third, we then excite the sample far enough away from the emission feature of interest so that both the excitation light and emission can be captured independent of each other. For instance, in the SiPcBu:PS spectra in **SI Figure 4.1** above, we excite the sample at 615 nm so that the excitation light goes back to the baseline before the onset of the emission around 650 nm. In addition, we also use excitation densities low enough (<<1 mW) such that we do not saturate the detector. Finally, for the reference sample, we made sure to use the same type of quartz substrate, cleaned, and with just the polystyrene host spray coated onto the substrate surface so that the only difference between reference and the sample of interest is the isolated sensitizer in the polystyrene film.

The equation used to calculate the PLQY is to take the ratio of the integrated counts over the entire emission spectrum with the difference between the integrated counts of the excitation peak with and without the sensitizer present. For instance in the SiPcBu:PS sample, we integrate the emission from 650 nm to 800 nm and integrate the excitation peak from 600 to 630 nm. The equation is:

$$PLQY = \frac{\int PL_{sens.}}{\int Exc._{blank} - \int Exc._{sample}} \tag{4.1}$$

5 Förster Resonance Energy Transfer Calculations and Relevant Plots

In order to quantify the energy transfer and the fate of the EET process postulated in the kinetic scheme in **Figure 3** and the photoluminescence data in **Figure 4** in the main text, we experimentally determined k_{FRET} for each sensitizer:PCBM film system. The impact of Förster resonance energy transfer (FRET) and Dexter transfer (DXT) on single molecules is well understood, providing a kinetic pathway for two dipole-coupled (FRET) or electronically-coupled (DXT) sensitizers to transfer excited state energy from one to the other under photoexcitation. The FRET rate constant, k_{FRET} is given the by the rate equation:

$$k_{FRET} = \left(\frac{R_0}{r_{DA}}\right)^6 \frac{1}{\tau_D} \tag{5.1}$$

where r_{DA} is the separation distance between the donor and acceptor, τ_D is the fluorescence lifetime of the donor, and R_0 is the Förster radius, given by the equation:

$$R_0^6 = 8.875 \times 10^{-5} \frac{\kappa^2 Q_D}{n^4} J \tag{5.2}$$

where, κ^2 is the orientation factor, Q_D is the fluorescence quantum yield of the donor, n is the refractive index, and J is the spectral overlap integral given as:

$$J = \int \hat{f}_D(\lambda)\epsilon_A(\lambda)\lambda^4 d\lambda \tag{5.3}$$

where $\hat{f}_D$ is the area normalized emission of the donor and ϵ_A is the molar absorptivity of the acceptor. In addition, if we assume that PET is governed by a Marcus formulation, given by:

$$k_{PET} = \frac{2\pi}{\hbar}|H_{DA}|^2 \frac{1}{\sqrt{4\pi\lambda k_B T}} exp\left[-\frac{(\lambda + \Delta G_{PET})^2}{4\lambda k_B T}\right] \tag{5.4}$$

and that the DXT rate, k_{DXT} is given as:

$$k_{DXT} = \frac{h}{2\pi}|H_{DA}|^2 J exp\left[\frac{-2r_{DA}}{L}\right] \tag{5.5}$$

where J is again the spectral overlap, r_{DA} is the donor-acceptor separation distance, and L is the sum of the van der Waals radii of the donor and acceptor molecules. Given that the orbital overlap $|H_{DA}|^2$ is the same as it is in **eqn. 5.4** for k_{PET}, then it is clear that k_{DXT} will be fast when k_{PET} is fast, so long as there is significant enough spectral overlap between the donor emission and acceptor absorption. Typically, exciton energy transfer (EET) out-competes photoinduced electron transfer (PET) in solution given the proximity of the donor (D) and acceptor (A). However, this process is not as well characterized for the solid-state or in three-dimensions, owing to the delocalization of the exciton and the possibility for longer range processes in the separate phases of D and A due to entropy (**need a reference here**).

In particular, we are interested in how the magnitude of k_{EET} in **eqn. 1** in the main text can account for the discrepancy in ϕ_{FC} as observed in **Figure 2** in the main text. We find that on average FRET is not fast enough ($k_{FRET,avg.} = 6.22 \times 10^{10}$ s^{-1}) to out-compete the PET rate constants, k_{FC} and k_{CT}, except in the normal region, and even then it only accounts for the discrepancy observed for SiPcBu (see **Table 2** in the main text for a comparison of the rate constants). Therefore, we conclude that FRET alone does not out-compete PET even when considering the entropy in a three-dimensional molecular system. Of note is that while we calculate the FRET rate constants and find that it is not fast enough to compete with PET except for very small ΔG_{CT} (> -0.2 eV), we can account for the discrepancy by including the Dexter transfer process, k_{DXT}.

To calculate k_{FRET}, we need a series of experimentally determined quantities: (1) τ_D, the fluorescence lifetime of each sensitizer, (2) Q_D, the fluorescence quantum yield of each sensitizer, (3) n, the index of refraction, (4) and $\hat{f}_D$, the area normalized emission of the donor, and ϵ_A, the molar absorptivity of the acceptor, both of which are used to calculate the spectral overlap, J. The fluorescence lifetime is determined with time-resolved photoluminescence measurements, which are shown below in **Figure 6.1**. The fluorescence quantum yield was determined in the previous section from **Figures 4.1-4.3**. The index of refraction was assumed to be the PCBM index of refraction, which is ca. 2 for a thin film.[9]. Finally, to calculate the spectral overlap we need the molar absorptivity which is given by the Beer-Lambert equation:

$$\epsilon = \frac{A}{cl} \tag{5.6}$$

where A is the absorbance, c is the concentration, and l is the path length. In this work, to find ϵ_A we took the absorption of neat PCBM and divided by the known concentration in the film (ca. 35 mM on average, as it changes slightly between each sensitizer:PCBM film) and the path length of the film, in this case the thickness of the spray coated films, which on average is 1.5 μm. We also need the area normalized emission spectrum of each sensitizer, which we can take from the previous photoluminescence measurements in **Section 1**. Combined together, these experiments are used to calculate k_{FRET} as described above in **eqn. 5.1**, the results of which can be found in **Figure S5.2** below in product with the number of microstates in the PCBM accepting host.

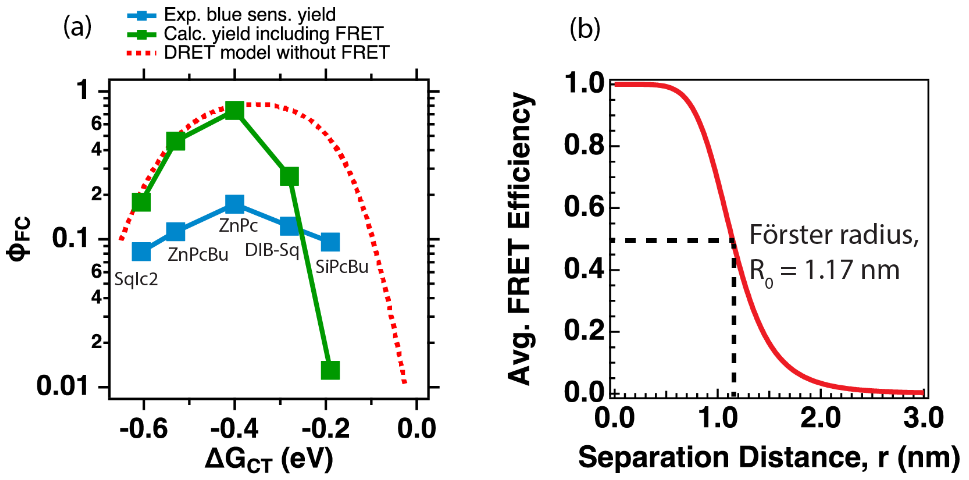

Figure S 5.1: **(a)** Free charge yield, ϕ_{FC} (assuming μ_e = 0.040 $cm^2V^{-1}s^{-1}$) for blue sensitizer data. The dashed red trace is ϕ_{FC} determined from the DRET model (parameters from Carr et al.) and assuming no FRET takes place (from **Eqns. 1** and **2** from the main text assuming k_{EET} is 0) while the green trace is ϕ_{FC} calculated from the DRET model with calculated FRET rate constants as a loss (from **Eqn. 5.1** and **Eqns. 1** and **2** from the main text assuming that k_{EET} is 6.22 × 10^{10} s^{-1}) for each sensitizer:PCBM film system. The blue trace is experimental ϕ_{FC} determined via TRMC. Each sensitizer is labeled corresponding to the sensitizer names in **Table 1** in the main text. **(b)** Average FRET efficiency calculated as a function of separation distance, r. The average Förster radius is denoted at 50% FRET efficiency inset on the image.

Figure 5.1a provides a comparison of the experimental FC yield and the calculated FC yield assuming that k_{EET} is 6.22 × 10^{10} s^{-1} to the DRET model which assumes competition for the excited state and no relevant energy transfer processes. In particular, we provide the calculated FC yield here for the intermediate case of only FRET, which were omitted from the main text for clarity. However, as was discussed in that section of the main text, the FC yield is only accounted for by FRET for SiPcBu, and partially for DIB-Sq, but is entirely inconsequential for the remaining sensitizers. This is attributed to the relative magnitude of the rate constants as shown in **Figure 5** in the main text. This direct comparison provided the evidence necessary that even when you consider FRET in a three-dimensional space and sum up over all possible microstates (see **Figure 5.2** below for k_{FRET} in 3D as a function of r) alongside the PET rate constants, it still does not compete effectively at most driving forces, instead we needed to consider the possibility of a faster Dexter process.

Figure 5.1b demonstrates the average FRET efficiency as a function of separation distance, r, where we find another key feature of the energy transfer process present in the blue sensitizers. In addition to the $k_{FRET,avg.}$ values discussed above, we also calculated the FRET efficiency and Förster radius, R_0 which is determined to be 1.17 nm. In addition, it is clear from the plot in **Figure 5.1b** that the FRET efficiency reaches ca. 0 by 3 nm, further corroborating that the energy transfer processes in the blue sensitizer molecular system results in short-range excitons in the PCBM host matrix within 3 nm of the donor species and is inside the quoted critical radius, r_c, from the DRET model (**Figure 3** and from Carr et al.[1]) where only CT states are generated. Therefore, the energy transfer from this system will

primarily produce bound CT states that do not go on to produce free charges and does not contribute to ϕ_{FC}.

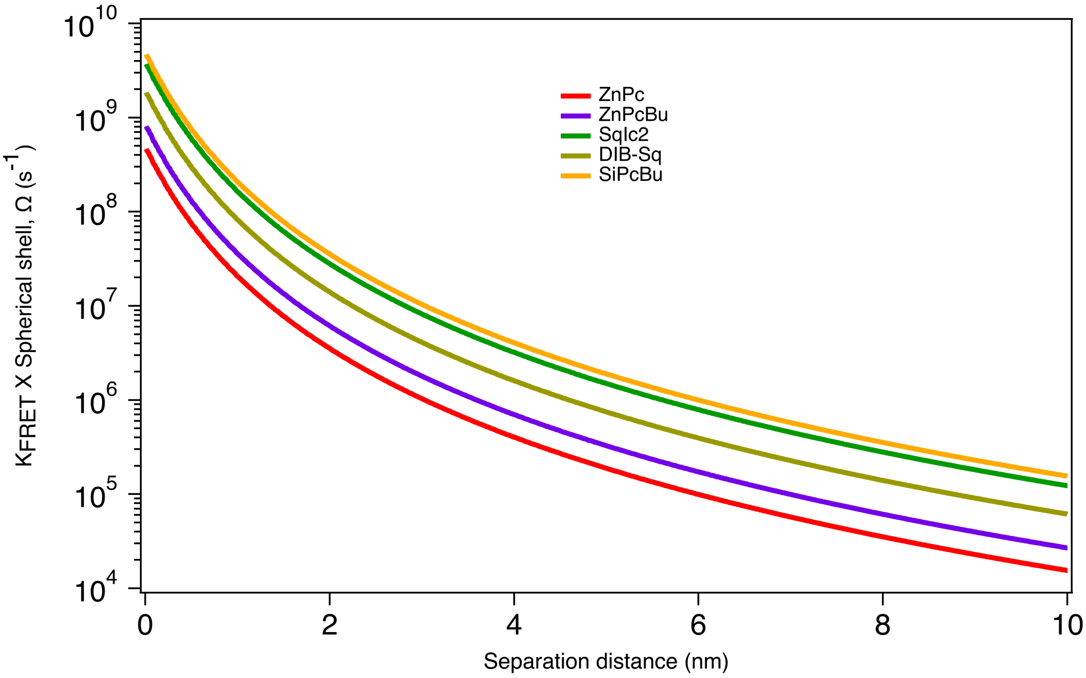

Figure S 5.2: k_{FRET} for each sensitizer in this work in product with the spherical shell of acceptor microstates to do energy transfer to in the PCBM accepting host, Ω, as it is described in Carr et al.

Figure 5.2 shows k_{FRET} in product with the total number of microstates in the PCBM accepting host to do energy transfer to. This quantity is then integrated over all r to find the total k_{FRET} for each sensitizer. The total k_{FRET} is the value that is averaged and used in the yield calculations in **Figure 5.1**. **Table 2** in the main text provides each averaged k_{FRET} value from these calculations.

6 Time-resolved Photoluminescence Transients with Fits and Parameters

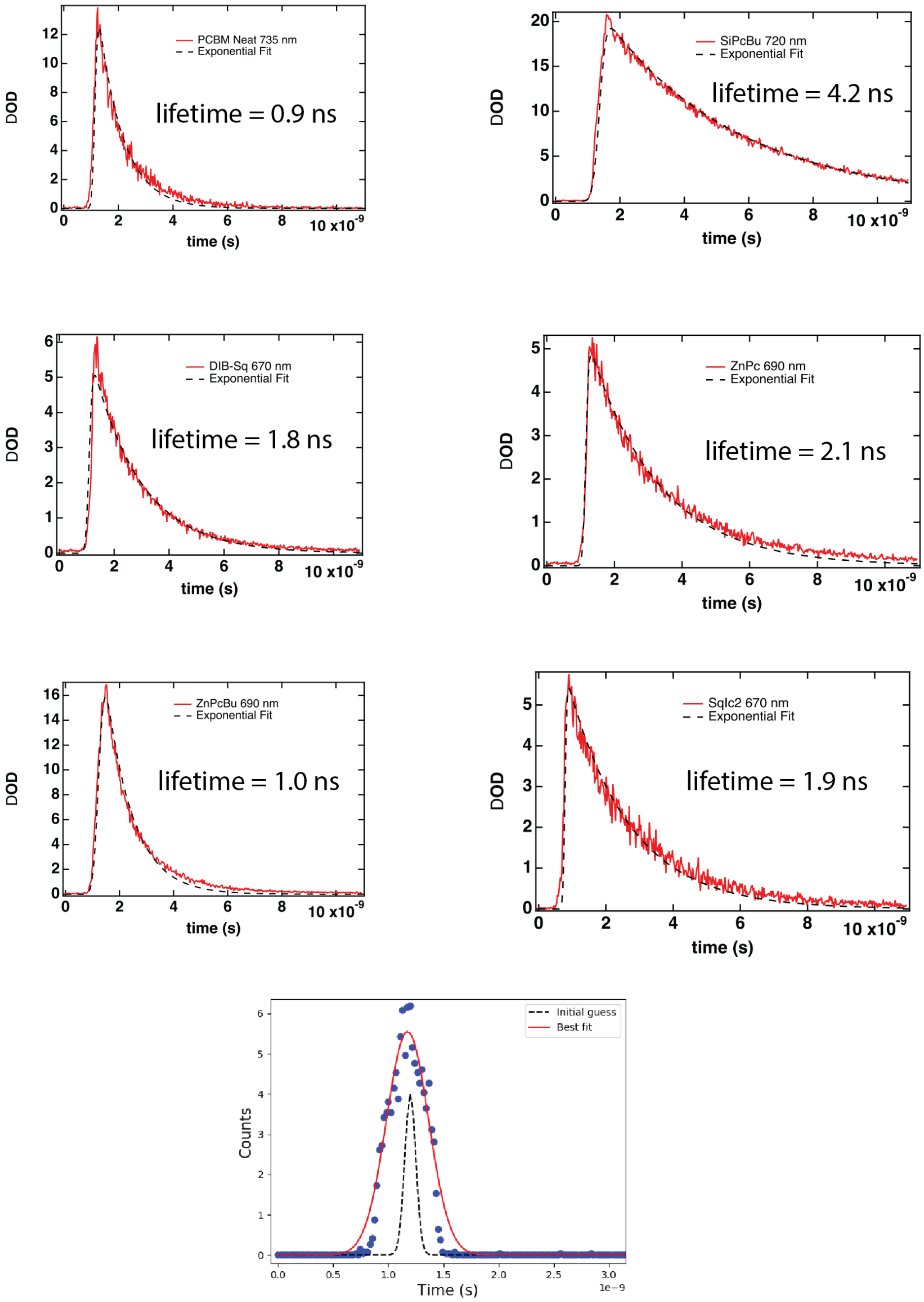

Figure S 6.1: TRPL transients collected for the sensitizer:PS spray coated film samples. Lifetimes are given inset on the each plot. Each black dashed line fit is a single exponential convolved with the gaussian instrument response function, shown in the final image at the bottom of the figure.

Table S 6.1: Table of all TRPL fitting parameters for PCBM and all sensitizers from the TRPL transients above. The IRF was collected once and kept constant across all samples, collected under the same excitation conditions at 650 nm.

Sample	IRF position (s)	IRF width (s)	τ_0 (s^{-1})	A_0
PCBM	1.15E-09	1.02E-10	1.11E+09	1
SiPcBu	1.15E-09	1.02E-10	2.41E+08	1
DIB-Sq	1.15E-09	1.02E-10	5.62E+08	1
ZnPc	1.15E-09	1.02E-10	4.68E+08	1
ZnPcBu	1.15E-09	1.02E-10	1.01E+09	1
SqIc2	1.15E-09	1.02E-10	5.41E+08	1

7 Transient Absorption Spectra and Excitation Dependent Experiments

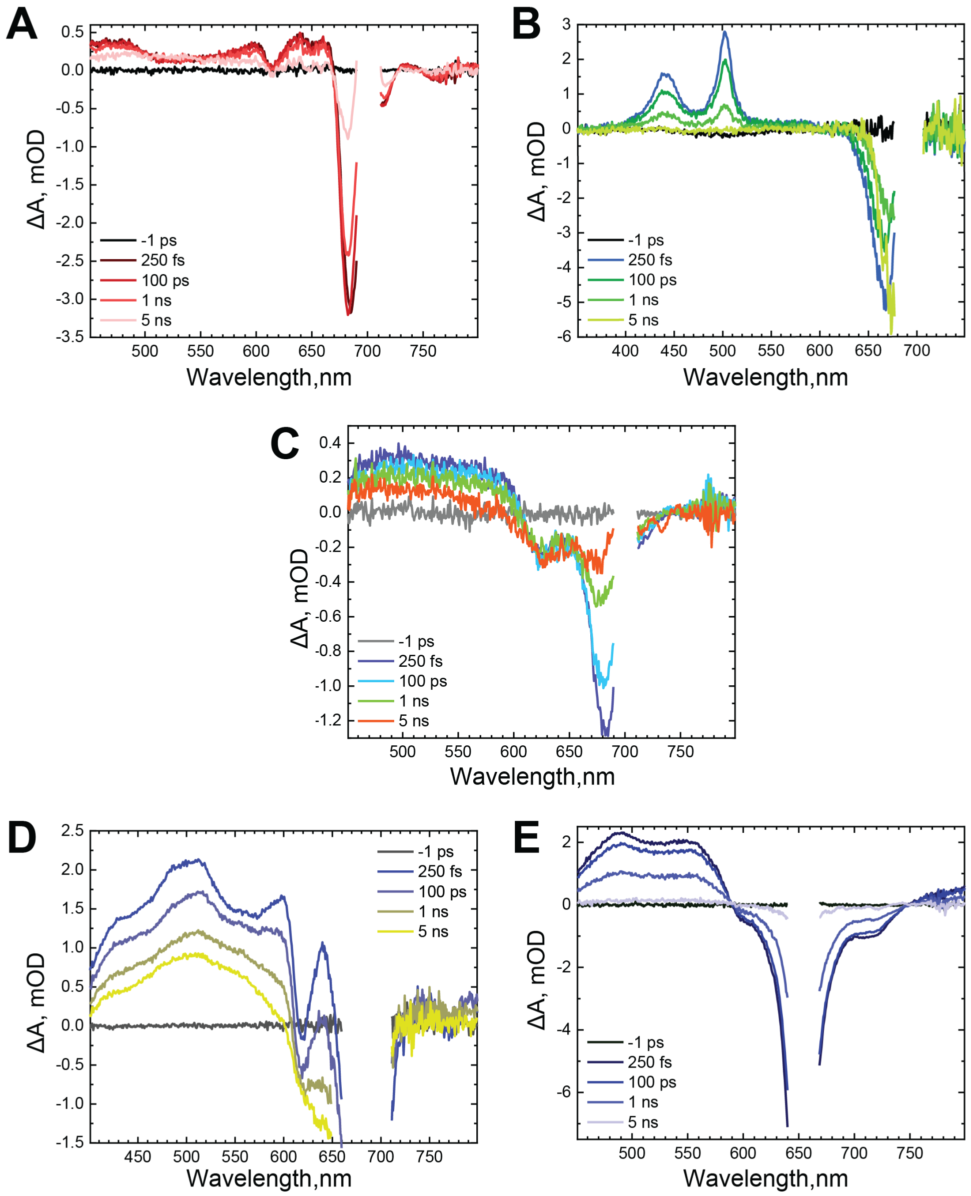

Figure S 7.1: Transient absorption spectra of **A.** SiPcBu, **B.** DIB-Sq, **C.** ZnPc, **D.** ZnPcBu, **E.** SqIc2 deposited via spray coating on quartz at 0.005 mol kg^{-1} in polystyrene and excited at either 700 nm (A, B, C, D) or 650 nm (E) at 150 nJ/pulse. The data are oriented in increasing driving force, with ZnPc (C) being the optimal driving force sample.

Upon photoexcitation, all samples contain a ground state bleach (GSB) and photoinduced absorptions (PIAs) with varying degrees of structure. Predictably, similar behavior is exhibited between the phthalocyanine donors, SiPcBu, ZnPC, and ZnPcBu (A,C,D), and the squaraine donors, DIB-Sq and SqIc2 (B, E). In the Pc series, intersystem crossing from the singlet to triplet excited state is evidenced through

evolution of the PIAs and changes in the GSB. The Sq series of donors exhibit excited state decay where the GSB and PIAs decay simultaneously and almost completely within the 5 ns window.

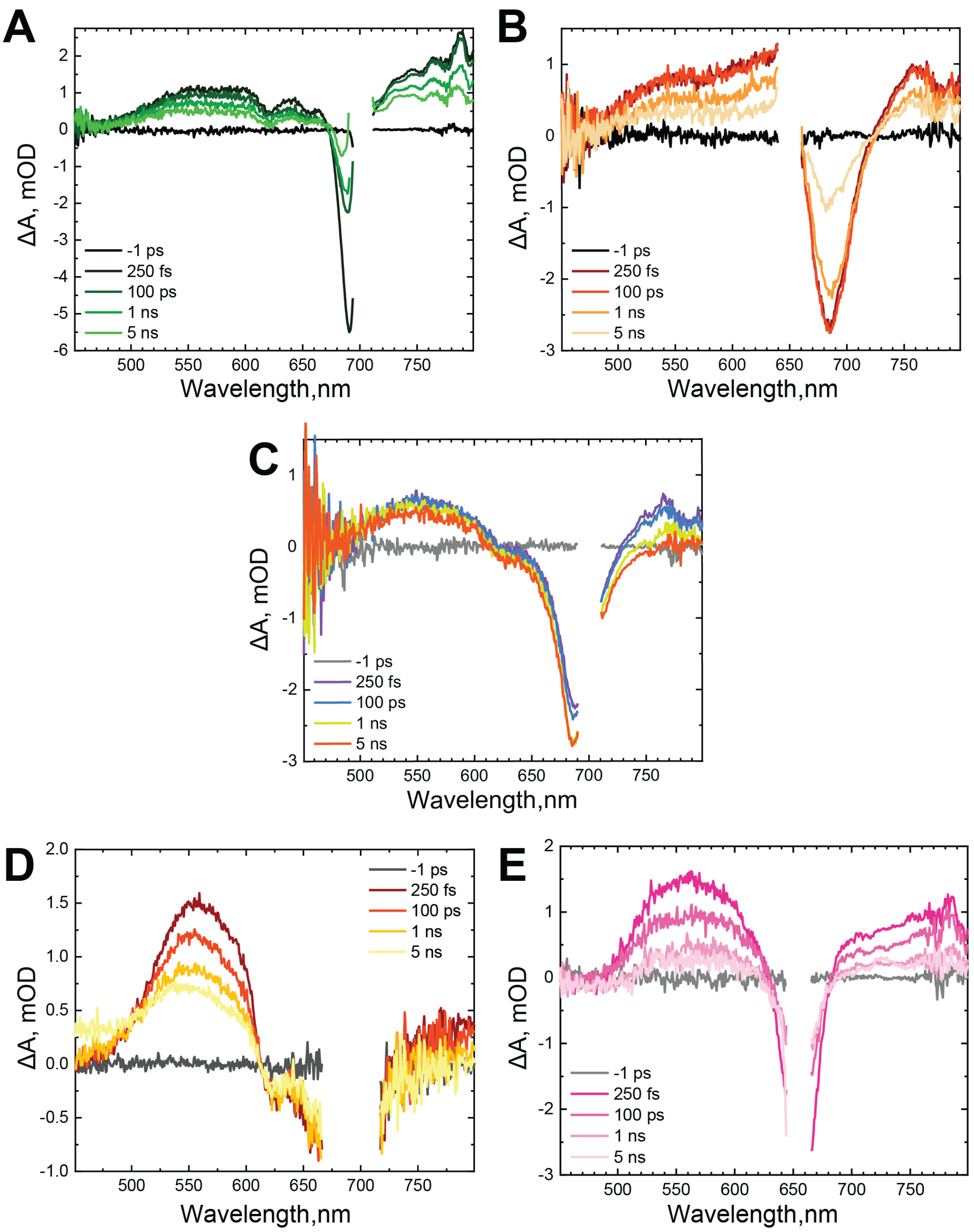

Figure S 7.2: Transient absorption spectra of **A.** SiPcBu, **B.** DIB-Sq, **C.** ZnPc, **D.** ZnPcBu, **E.** SqIc2 deposited via spray coating on quartz at 0.005 mol kg^{-1} in the PCBM accepting host and excited at either 700 nm (A, B, C, D) or 650 nm (E) at 150 nJ/pulse. The data are oriented in increasing driving force, with ZnPc (C) being the optimal driving force sample.

Unlike the isolated donors in polystyrene, where behavior is grouped based on chemical identity, the blends exhibit a trend based on driving force. The lowest

driving force case, SiPcBu, contains spectral features from both the SiPcBu and the PCBM acceptor. As described in the manuscript, the GSB of SiPcBu decays much more rapidly in the case of the blend than in polystyrene consistent with singlet energy transfer to the PCBM host. This is not quantitative and a portion of the SiPcBu molecules undergo ISC with similar behavior to the SiPcBu in PS case. In DIB-Sq, the GSB is assigned to the donor, but the PIA features are a combination of the PCBM (**Figure 7.4**) and a new absorption feature. The bleach of the donor, DIB-Sq, has a longer lifetime than in polystyrene, and there is no evidence of the excited state absorption features assigned to isolated DIB-Sq. Therefore, the new PIA feature can only be assigned to an interaction between donor and acceptor. Based on other data presented in this figure and main text, we can assign a small portion of charge separated states. In the optimal driving force case, ZnPc, we again observe a GSB due to the donor and a PIA, but over the course of 5 ns there is minimal change in the spectrum. This is indicative of a long-lived charge separated state and correlates well with the TRMC observation of increased free charge generation at the optimal driving force. The decay in the red PIA (>750nm) is due to parasitic absorption of the PCBM host, which results in a small growth of the ZnPc GSB with a time constant of approx. 300 ps. We also looked at the transient absorption data for the ZnPc blend when exciting at 600nm (**Figure S7.3**), where a majority of the photons are absorbed by the PCBM. There is a striking difference in behavior, where we observe the ZnPc GSB continuing to grow in amplitude over the 5 ns window concomitant with a decay in the red PIA from the PCBM, while the PIA centered at 550 nm remains relatively constant. The multiexponential nature of this growth is consistent with a PCBM excited state diffusing to a ZnPc interface and undergoing charge separation. These data are in agreement with the TRMC data.The higher lying driving force cases, ZnPcBu and SqIc2 exhibit similar spectral features and kinetic behavior despite chemical structure differences. In SqIc2, the PCBM-like excited state feature along with the GSB of the dilute donor decay together with a 100 ps lifetime, which suggests the primary mechanism of decay is CT state formation.

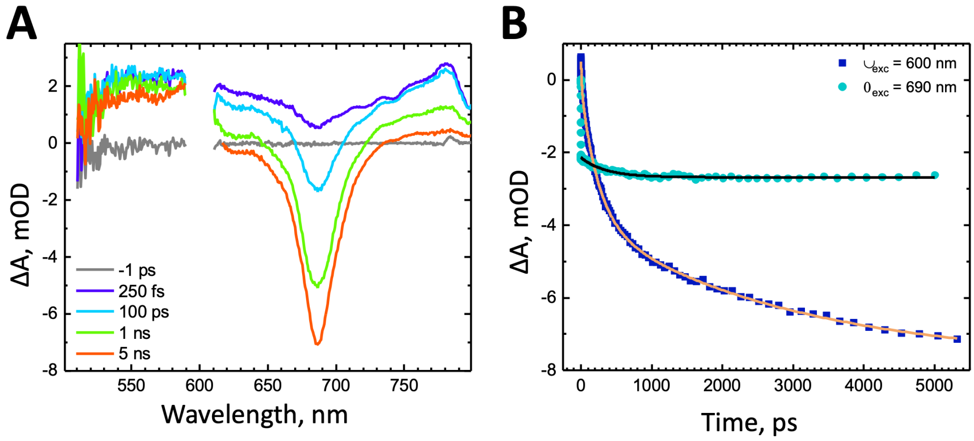

Figure S 7.3: A. Transient absorption spectra of ZnPc deposited via spray coating on quartz at 0.005 mol kg^{-1} in the PCBM accepting host excited at 600 nm at 150 nJ/pulse. **B.** Comparison of GSB kinetics (680 nm) of ZnPc in PCBM accepting host at excitation wavelengths of 600 nm (dark blue squares) and 690 nm (teal circles). The magnitude of growth of the GSB of ZnPc when exciting the PCBM host is much larger than exciting into the dilute donor. Consistent with the TRMC measurements, these data show that excitation directly into the PCBM host creates more charge separated states.

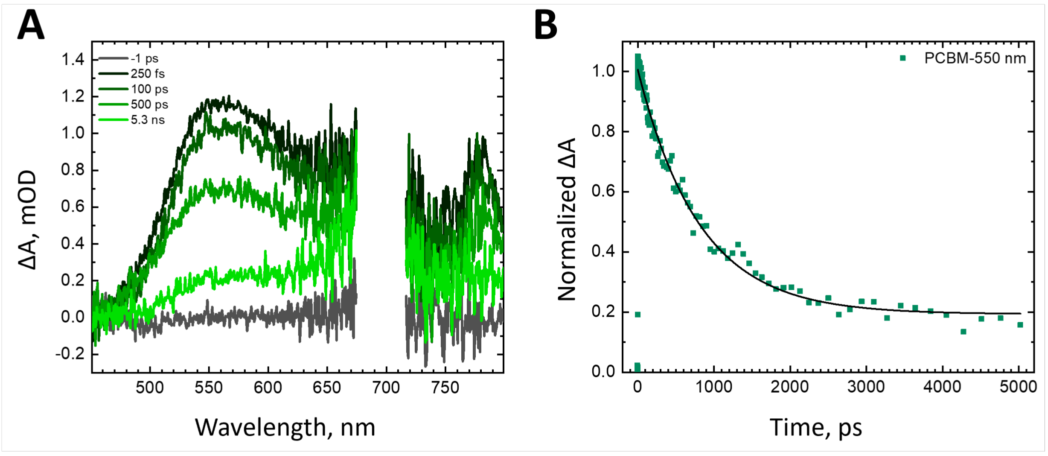

Figure S 7.4: A. Transient absorption spectra of PCBM deposited via spray coating on quartz excited at 700 nm at 150 nJ/pulse. **B.** Kinetic trace monitored at 550 nm, the PCBM PIA. The data follow a simple exponential decay (shown as black line) with a excited state decay lifetime of 800 ps.

8 Do Triplet States Quench Free Charge Yield?

In the main text we concluded that the reason the "blue sensitizers" exhibit lower charge yield than the "red sensitizers" is that energy transfer from the "blue sen-

sitizers" out-competes free charge generation. The alternative hypothesis that we consider here is that a triplet state might be quenching the production of free charges in one of two ways. First, it could be that ISC on the PCBM after EET is fast enough to compete with charge transfer, leading to PCBM triplets (and possibly back Dexter transfer to sensitizer triplets) as the loss mechanism. Second, the triplet energy of these "blue sensitizers" may be closer to the CT state manifold, and thus accelerate geminate recombination on a timescale fast enough (sub-nanosecond) to reduce ϕ_{FC}. These alternatives can be ruled out via consideration of the literature, and certain aspects of our experimental data. We discuss them in order below.

PCBM has a high triplet yield and an ISC rate on the order of 1×10^9 s^{-1}.[10] However, intersystem crossing can be ruled out as a reason that free-charge generation might turn off after EET from the sensitizer to the PCBM, as this is not consistent with the large free charge yield we observe when the PCBM is excited directly.

The second hypothesis requires somewhat more work to explain, and ultimately falsify. The triplet energy of ZnPc is ca. 1.2 eV[11] while the triplet energy of PCBM lies close to its singlet energy at ca. 1.5 eV[12]. In comparison, we estimate that the CT state energies in our experiments extend from about 1.5 eV down to 1 eV, taking the exciton energy as 1.7 eV and $E_{CT} = E_{Ex} - \Delta G_{CT}$. The FC states may lie as much as 0.3-0.4 eV above the nearest-neighbor CT states. Thus, the triplet state of the phthalocyanine, and possibly the PCBM, both lie at energies bracketed by the CT and FC states we consider. In such a case one might expect to have a fast Marcus-like recombination process from the higher lying FC states through the triplet of the phthalocyanine, ultimately resulting in a reduction of the free charge population.[13] However, both the TA and the TRMC data rule this process out as a major loss pathway. Production of a phthalocyanine triplet would result in a well-known excited-state spectrum, which we do not observe in significant quantity. As noted in the main text, the sensitized PCBM films exhibit excited-state spectra at 5 ns that cannot be explained by a linear combination of PCBM and sensitizer features in any of our samples. Moreover, this behavior would also be expected to produce a ΔG_{CT} dependence of the lifetime of the free charges we observe via TRMC. There is no evidence of such systematic lifetime variations in our TRMC data (**SI Section 3** and **SI Figures 4.1-4.6** and **SI Table 4.1**). Instead, it seems that the free charge recombination present in our system is dominated by diffusive encounter probabilities of the free electron with holes, and is not able to account for the drop in ϕ_{FC} observed in **Figure 2**.

9 Do Delocalized Excitons Explain Yield Enhancements Observed for Direct PCBM Excitation?

In the main text we describe an *enhancement* in free charge yield when the PCBM is directly excited between 565 and 640 nm, above the energy of the sensitizer, shown in **Figure Figure 2a**. Our main hypothesis is that a diffusing PCBM exciton is able to sample the full configuration of microstates, allowing long-range electron transfer and free charge generation, whereas direct excitation of the sensitizer followed by EET to the PCBM confines electron transfer to the nearest-neighbor pair. Here, we consider an alternative hypothesis that could explain this enhancement. It is possible

that EET to the PCBM produces the most relaxed Frenkel exciton, whilst direct excitation with higher photon energies creates a hot delocalized PCBM exciton with a higher propensity to produce free charges upon electron transfer. Two experiments provide evidence that falsify this hypothesis.

First, we conducted a TA experiment at 600 nm excitation on the ZnPc:PCBM sample (see **SI Figure 7.3b**) to compare the ground state bleach (GSB) kinetics of ZnPc depending on whether excitation is primarily into the PCBM host or directly into the sensitizer, which can only occur in response to charge transfer considering both the energetics of the system and the preponderance of free-charge generation observed via TRMC. When the PCBM host is excited at 600 nm the GSB signal grows slowly over the 5 ns time window. In contrast, delocalized PCBM excitons generated at 600 nm would be expected to relax to the most localized exciton within a few ps. Thus, we do not believe the probable lifetime of such a state is consistent with the charge generation kinetics we observe.

Second, we conducted a TRMC control experiment on the ZnPc:PCBM film exciting at the lowest possible energy: 720 nm, right at the PCBM absorption onset, below the absorption of the ZnPc(see **SI Figure 3.7** for transients and yield-mobility product). Excitation into the ZnPc:PCBM film at 720 nm results in the same free-charge yield (within error) as the excitation at 640 nm, both of which are about 3.7x higher than the yield when the ZnPc sensitizer is directly excited at 680 nm. This observation is similarly inconsistent with the hypothesis that the delocalized, "hot" exciton in PCBM is enhancing free-charge yield, as excitations directly into the localized PCBM exciton result in the same free-charge yield as those at higher energy. Together with the TA results discussed above, we argue these experiments rule out any contribution from "hot" PCBM excitons.

References

(1) Carr, J. M.; Allen, T. G.; Larson, B. W.; Davydenko, I. G.; Dasari, R. R.; Barlow, S.; Marder, S. R.; Reid, O. G.; Rumbles, G. Short and long-range electron transfer compete to determine free-charge yield in organic semiconductors. *Materials Horizons* **2022**, DOI: 10.1039/d1mh01331a.

(2) Ferguson, A. J.; Kopidakis, N.; Shaheen, S. E.; Rumbles, G. Quenching of excitons by holes in poly(3-hexylthiophene) films. *Journal of Physical Chemistry C* **2008**, *112*, 9865–9871.

(3) Reid, O. G.; Rumbles, G. Quantitative transient absorption measurements of polaron yield and absorption coefficient in neat conjugated polymers. *Journal of Physical Chemistry Letters* **2013**, *4*, 2348–2355.

(4) Hodgkiss, J. M.; Albert-Seifried, S.; Rao, A.; Barker, A. J.; Campbell, A. R.; Marsh, R. A.; Friend, R. H. Exciton-charge annihilation in organic semiconductor films. *Advanced Functional Materials* **2012**, *22*, 1567–1577.

(5) O'Connor, M. M.; Aubry, T. J.; Reid, O. G.; Rumbles, G. Charge Concentration Limits the Hydrogen Evolution Rate in Organic Nanoparticle Photocatalysts. *Advanced Materials* **2023**, 2210481.

(6) Ferguson, A. J.; Kopidakis, N.; Shaheen, S. E.; Rumbles, G. Dark carriers, trapping, and activation control of carrier recombination in Neat P3HT and P3HT:PCBM blends. *Journal of Physical Chemistry C* **2011**, *115*, 23134–23148.

(7) De Haas, M. P.; Warman, J. M.; Anthopoulos, T. D.; De Leeuw, D. M. The mobility and decay kinetics of charge carriers in pulse-ionized microcrystalline PCBM powder. *Advanced Functional Materials* **2006**, *16*, 2274–2280.

(8) Brouwer, A. M. Standards for photoluminescence quantum yield measurements in solution (IUPAC technical report), 2011.

(9) Moul´, A. J.; Meerholz, K. Interference method for the determination of the complex refractive index of thin polymer layers. *Applied Physics Letters* **2007**, *91*, 061901.

(10) Chow, P. C.; Albert-Seifried, S.; Gélinas, S.; Friend, R. H. Nanosecond intersystem crossing times in fullerene acceptors: Implications for organic photovoltaic diodes. *Advanced Materials* **2014**, *26*, 4851–4854.

(11) Savolainen, J.; van der Linden, D.; Dijkhuizen, N.; Herek, J. L. Characterizing the functional dynamics of zinc phthalocyanine from femtoseconds to nanoseconds. *Journal of Photochemistry and Photobiology A: Chemistry* **2008**, *196*, 99–105.

(12) Di Nuzzo, D.; Aguirre, A.; Shahid, M.; Gevaerts, V. S.; Meskers, S. C.; Janssen, R. A. Improved film morphology reduces charge carrier recombination into the triplet excited state in a small bandgap polymer-fullerene photovoltaic cell. *Advanced Materials* **2010**, *22*, 4321–4324.

(13) Chow, P. C.; Chan, C. C.; Ma, C.; Zou, X.; Yan, H.; Wong, K. S. Factors That Prevent Spin-Triplet Recombination in Non-fullerene Organic Photovoltaics. *Journal of Physical Chemistry Letters* **2021**, *12*, 5045–5051.
